# International survey of people living with chronic conditions (PaRIS survey): effects of general practitioner non-participation on the representativeness of the Norwegian patient data

**DOI:** 10.1186/s12913-024-11751-0

**Published:** 2024-10-18

**Authors:** Oyvind Bjertnaes, Kjersti E. Skudal, Michael J. van den Berg, Ian Porter, Olaf Holmboe, Rebecka M. Norman, Hilde H. Iversen, Lina H. Ellingsen-Dalskau, Jose M. Valderas

**Affiliations:** 1 Department of health services research, Division for health services, Norwegian Institute of Public Health, Oslo, 0473 Norway; 2 Department for quality indicators and user surveys, Health Directorate, Oslo, Norway; 3 Directorate for Employment, Labour and Social affairs, Organisation for Economic Co-operation and Development, Paris, France; 4 Health Services and Policy Research Group, University of Exeter Medical School, Exeter, UK; 5 Department of Family Medicine, National University Health System, Level 9, Singapore, Singapore

**Keywords:** General practice, General practitioner, Health care surveys, Patient satisfaction, Physician-patient relations, Quality improvement

## Abstract

**Background:**

The International Survey of People living with Chronic Conditions (OECD-PaRIS survey), aims to systematically gather patient-reported experiences (PREMs) and - outcomes (PROMs) and potential predictors for these outcomes for persons with chronic conditions as well as information from professionals about health care provided. In such patient surveys, the advantages of a multilevel (nested) approach in which patients are sampled ‘within providers’ need to be balanced against the potential for bias if patient populations from participating GPs significantly differ from those of non-participating GPs. The objective was to assess the effects of general practitioner (GP) non-participation on the representativeness of the Norwegian patient data of the International Survey of People living with Chronic Conditions (OECD-PaRIS survey).

**Methods:**

To test all aspects of the first main PaRIS survey, it was preceded by a field trial which this paper reports on the Norwegian part of. For the Norwegian part of the field trial in 2022, we randomly sampled and surveyed 75 GPs and 125 patients 45 years and older for each GP, regardless of whether their GP were also participating in the study. GPs were sampled from a national register that included all GPs. The surveys were primarily digital, but we sent postal questionnaires to non-digital patients and non-responding digital patients. We compared GP and patient characteristics as well as patient-reported experiences and outcomes according to GP participation status in bivariate analysis, supplemented with multiple linear regressions with PREMs/PROMs as dependent variables and participation status as independent adjusting for significant patient factors.

**Results:**

17 of 75 sampled GPs participated (22.7%), of which 993 of 2,015 patients responded (49.3%). 3,347 of 7,080 patients of non-responding GPs answered (47.3%). Persons with chronic conditions from participating GPs reported significantly better patient-centred coordinated care (*p* = 0.017), overall experiences with the GP office the last 12 months (*p* = 0.004), mental well-being (*p* = 0.039) and mental health (*p* = 0.013) than patients from non-participating GPs. The raw differences between participating and non-participating GPs on patient-reported experiences and – outcomes varied from 1.5 to 2.9 points on a 0-100 scale, and from 2.2 to 3.0 after adjustment for case-mix.

**Conclusions:**

The Norwegian field trial indicates that estimates based on participants in the PaRIS survey may modestly overestimate patient-reported experiences and -outcomes at the aggregated level and the need for more research within and across countries to identify and address this potential bias.

**Supplementary Information:**

The online version contains supplementary material available at 10.1186/s12913-024-11751-0.

## Background

The number of persons with chronic conditions and multimorbidity is increasing globally, with negative consequences for quality of life, the ability to work and healthcare costs [1]. The International Survey of People Living with Chronic Conditions (PaRIS survey) is a large and novel initiative by the OECD, which combines a provider survey with a large-scale patient survey, aiming to estimate population values for patient-reported experiences and - outcomes and using the provider survey and aggregated system level information to explain variation in patient reported experiences and outcomes (PREMs and PROMs). PaRIS aims to help policymakers understand how their health system addresses the needs of people living with chronic conditions and help countries to make their health systems more responsive, and fill a significant gap in knowledge by providing systematic internationally comparable data on the healthcare outcomes and experiences as reported by patients [2]. The PaRIS initiative originated in 2017 when the Health Ministers of the OECD member states mandated OECD to lead the development and implementation work, with 21 countries across Europe, USA, Australia and Asia committing to implement the survey [2].

To achieve valid comparisons across countries, standardisation of instruments and procedures for sampling and data collection are key elements in international health care surveys [2, 3].

In contrast to population-based surveys, the PaRIS survey has a multilevel sampling design with patients nested within primary care providers (PCPs). Both PCPs and their patients fill out questionnaires, capturing information about the characteristics of and services provided and patient-reported outcomes and care experiences respectively. An advantage of this approach is that patient-reported measures could be linked with practice characteristics, which provides opportunities for identifying care models and factors on the provider level that might be associated with better outcomes and experiences. In most countries, the standard is to first recruit PCPs and then patients from these providers [2]. This two-step sampling approach has been used in similar international research previously, but getting PCPs to participate is a major challenge: e.g., participation rates were less than 20% for eleven of 34 countries with valid numbers in a previous international health care survey [4]. Although the multilevel design has clear advantages for the identification of relevant practice level variables that might affect patient outcomes and experiences, this design could also create systematic bias if provider characteristics vary between participants and non-participants and these characteristics are related to survey outcomes. For example, specialists in general practice have significantly better patient experience scores than non-specialists [5], which means that patient experience estimates will be biased if the proportion of specialists differs between participants and non-participants.

The PaRIS survey is a new initiative and most Norwegian GPs had no prior knowledge about the survey. Sending an invitation letter to GPs to participate in an international survey about chronic health care might result in a higher probability of recruiting GPs with more interest in this topic and/or with better chronic care performance. This is because what is made salient in the survey approach and how much the person negatively or positively values the attribute(s) are important for the decision to refuse or accept [6, 7]. The title, purpose and content of the survey relate to health services for persons with chronic conditions, which with necessity will be visible and salient in the invitation letter to GPs. The decision to participate in a survey is normally made quickly [6], and the saliency of chronic care in the invitation letter might especially attract GPs with an interest in chronic care to participate.

Given accessibility to all relevant background variables for participants and non-participants, this challenge could partly be handled with non-response analysis and weighting. However, the accessibility to data about non-participants is often scarce in international health care surveys, with non-response weighting only conducted with a limited number of variables with available population distributions or not at all [4, 8–10].

In high-stake international initiatives like the PaRIS survey, the presence and amount of bias should be thoroughly assessed and corrected as much as possible. Many sources of survey bias will be assessed as part of the standard data analysis of the international survey (e.g., differential item functioning across countries), but in-depth analysis of non-response bias and coverage bias is less straightforward. In most countries opportunities for this are limited, since national registries of primary care patients are not available or not accessible. In contrast to most other countries, Norway has a national register with patients nested within providers (GPs), covering all regular GPs in Norway and more than 99% of the population. The register includes background variables for both participating and non-participating GPs and patients, and meant that patients could be randomly selected and included regardless of provider participation in the survey. This register provides an opportunity to analyse differences in PREMS and PROMS between patients of GPs not responding to the survey (non-participants) and those responding (participants).

The objective of this study was to assess the effects of GP non-participation on the representativeness of the Norwegian sample, by comparing GP and patient characteristics and patient-reported experiences and outcomes between participating and non-participating GPs and their respective patients.

## Methods

### Setting

All residents in Norway are entitled to a regular GP, and around 99% of the population are part of the regular GP scheme [11]. Norwegian GPs are gatekeepers for the national insurance scheme, and patients are referred from a GP to specialised medical care when needed. The GP practices are, in general, small units with 2–5 GPs, most of which are self-employed [12]. In Norway, a competence regulation demands that all GPs entering the regular GP scheme after 1st of March 2017 are specialists in general practice or under training to be specialists, with some exceptions because of recruitment challenges.

### Sampling and register data

We used the national GP register as a sample frame. The register included all regular GPs in Norway (around 5,000), with patients nested within GPs and covering almost the whole population in Norway. GPs on leave of absence were excluded. Patients 45 years or older with at least one GP consultation in the six months preceding the sampling were included, in line with the PaRIS protocol [2]. The OECD and their research consortium calculated the sample size for the field trial, aiming at 25 participating GPs/providers and minimum 50 responding patients from each of these in Norway. We randomly selected 75 GPs and 125 patients for each GP, or all patients if the number of patients was lower than 125. The national GP register included name and contact information for GPs and patients, and additional data on GPs, such as age, gender, type of practice (solo/group), whether specialist in general medicine or not, and the number of patients on the list. To obtain additional data on patients, such as diagnosis and the number of consultations the last two years, we gathered data from the Norwegian Control and Payment of Health Reimbursements Database.

### Survey data collection

Data collection took place from May 2022 to August 2022. Patients registered in the national digital portal Helsenorge.no (84.6%) were sent a digital invitation and up to three reminders, the last a postal reminder with a pen-and-paper questionnaire included. Patients without a registered user in Helsenorge.no received a postal invitation with a digital response option, and one reminder with a pen-and-paper questionnaire included. GPs with a user in Helsenorge.no (92%) were sent a digital invitation to participate and up to two reminders, the latter a postal invitation letter. GPs without a user in Helsenorge.no were sent a postal invitation to participate and one postal reminder.

Facilitated by the national register with patients nested within GPs, we included patients regardless of GP participation in the Norwegian field trial. GP and practice characteristics available from the national GP register included gender, age, specialist status, mean years as GP, size of practice patient list, available spots on practice patient list (difference between agreed list size and number of patients on the list), type of practice, whether practices had an independent patient list or shared with other GPs. Thus, we were able to compare GP characteristics, patient characteristics and PREMs and PROMs for persons with chronic conditions between participating and non-participating GPs.

### Questionnaires

The development of the conceptual model and questionnaires for the PaRIS survey has been described elsewhere [13–15]. The patient questionnaire in the field trial consisted of 121 items, mainly based on existing validated instruments about patient-reported experiences (PREMs), patient-reported outcomes (PROMs) and potential predictors like health and health care capabilities, health behaviors and individual and sociodemographic factors. The GP questionnaire consisted of questions about health care provided for persons with chronic conditions, but apart from response status the GP survey was not part of this study.

Source questionnaires in English are provided as Appendix 1–2.

### Outcome measures

Of all the patient reported measures (validated and new scales and sets of adapted and new items) in the field version of the survey, we selected a PREM measure that was developed and tested among persons with one or more chronic conditions, the Patient-Centred Coordinated Care Experience Questionnaire (P3CEQ) [16, 17]. The P3CEQ consists of 10 items measuring person-centred care and care coordination, with two items being dichotomous (having a responsible health personnel or not; having a care plan or not) and the rest with multiple and varying response categories (mostly “Not at all” to “Always”). We retained all 10 original items and thus preserved the content validity of the instrument, by creating an index, in line with expectations for formative constructs [18]. All items were recoded to 0 or 1, the latter being the positive response option (“yes”) for items with binary response options and the two most positive response options for the remaining items. Construct validity of the P3CEQ index was confirmed by small significant correlations with the WHO-5 scale and the two-item PROMIS^®^-scales [19].

We also selected two short generic PROMs measures: the WHO-5 Well-Being Index [20] and the two-item PROMIS^®^ Global Physical and Mental Health scales [21]. All items in the WHO-5 and PROMIS^®^-scales were linearly transformed to a 0-100 scale, where 100 is the best outcome. For the WHO-5, average scores across items were computed for all persons having at least three (of five) valid answers, while PROMIS scale scores were computed for persons having one (of two) valid answer for each scale. We confirmed the psychometric properties of the scales (Cronbach’s alpha > 0.7 for all scales).

Finally, we selected an item on the overall evaluation of the GP office in the previous 12 months. The item about overall evaluation of the GP office had five response categories, ranging from “Excellent” to “Poor”. The item was linearly transformed to a 0-100 scale, where 100 is the best outcome.

### Statistical analysis

We compared GP characteristics, patient characteristics and PREMs and PROMs for persons with chronic conditions between participating and non-participating GPs, using Pearson’s chi-square for categorical variables and t-tests for continuous variables. The latter was supplemented with multiple linear regressions with PREMs and PROMs scales/index as dependent variables and participation status as independent, controlling for significant patient factors (age, gender) from the bivariate analysis.

## Results

17 of 75 (22.7%) sampled GPs participated (Table 1). None of the GP factors or practice factors differed significantly between participating and non-participating GPs (Table 1). Among those patients whose GPs agreed to participate 993 of 2,015 (49.3%) responded, while 3,347 of 7,080 (47.3%) patients answered for those with non-responding GPs.

**Table 1:** Comparisons of background variables between participating and non-participating GPs

	Participating GPs (*n* = 17)	Non-participating GPs (*n* = 58)	*P* value
*GP factors*:			
Women, % (n)	35.3 (6)	34.5 (20)	0.951
Mean age (sd)	49.3 (11.1)	50.3 (10.7)	0.742
Specialist in general practice, % (n)	76.5 (13)	70.7 (41)	0.641
Mean years as GP (sd)^a^	14.1 (8.2)	13.7 (7.5)	0.868
*Practice factors*:			
Mean number of patients on the list (sd)	1,027.2 (282.9)	1,006.4 (391.6)	0.839
Mean number of available spots on patient list (sd)	15.2 (101.8)	23.9 (106.5)	0.766
Group practice, % (n)	94.1 (16)	94.8 (55)	0.909
GPs with independent patient list, % (n)	94.1 (16)	93.1 (54)	0.883

Among all patients regardless of patient participation status, those with participating GPs there were significantly more women (*p* < 0.001), and had higher age (*p* = 0.024) and more consultations (*p* < 0.001) and diagnoses (*p* < 0.001) than those with non-participating GPs (Table 2). The respondent sample for participating GPs had significantly more women (*p* = 0.032), fewer patients living in rural areas (*p* < 0.001), and more consultations (*p* = 0.011) and diagnoses (*p* < 0.001) than non-participating GPs.

**Table 2:** Comparisons of patient characteristics between participating and non-participating GPs, total sample and respondent sample

	Patients from participating GPs	Patients from non-participating GPs	*P* value
Study populations’^a^ part of total patient list, %	37.0	37.1	0.559
45 years and older, part of total patient list, %	47.5	49.1	<0.001
*Total patient sample*:			
N	2,015	7,080	-
Women, % (n)	57.3 (1,155)	52.5 (3,715)	<0.001
Mean age (sd)	64.8 (12.1)	64.1 (12.2)	0.024
Mean number of consultations last 24 months (sd)	16.8 (15.7)	15.2 (15.3)	< 0.001
Mean number of diagnoses last 24 months (sd)	6.4 (3.9)	5.3 (3.5)	<0.001
Mean years of connection to GP (sd)	10.4 (7.6)	10.2 (7.8)	0.316
*Respondent sample*:			
n (%)	993 (49.3)	3,347 (47.3)	0.112
Women, % (n)	56.7 (563	52.8 (1,768)	0.032
Mean age (sd)	65.5 (11.1)	65.0 (11.2)	0.160
Mean number of consultations last 24 months (sd)	16.2 (14.6)	14.9 (13.3)	0.011
Mean number of diagnoses last 24 months (sd)	6.3 (3.8)	5.3 (3.4)	<0.001
Mean years of connection to GP (sd)	10.6 (7.5)	10.7 (7.8)	0.924
One or more self-reported chronic conditions, % (n)	75.2 (747)	78.0 (2,612)	0.063
Mean number of self-reported chronic conditions (sd)	1.44 (1.23)	1.51 (1.25)	0.121
Born in Norway, % (n	92.4 (912)	91.6 (3,035)	0.426
Net family income 56,000 NOK or more, % (n)	30.3 (243)	29.5 (790)	0.782
University degree, % (n)	43.1 (421)	41.3 (1,355)	0.310
Living in rural areas	31.9 (314	41.1 (1,354)	<0.001

^a^Study population: persons 45 years and older with at least one consultation last six months

Persons with chronic conditions from participating GPs reported significantly better patient-centred coordinated care (*p* = 0.017), overall experiences with the GP office the last 12 months (*p* = 0.004), well-being (*p* = 0.039) and mental health (*p* = 0.013) than patients from non-participating GPs (Table 3). Scores on the PROMIS-physical scale did not differ significantly between persons with chronic conditions from GP participants and non-participants (*p* = 0.083), but the item about overall physical health had significantly different scores (*p* = 0.010). For the primary outcomes, the raw differences ranged from 1.5 (PROMIS-physical) to 2.9 points (overall evaluation of GP office) on a 0-100 scale, with slightly larger differences on PREMs compared to PROMs. Multiple linear regression models showed that the differences for the primary outcomes were at the same level and significant after controlling for significant patient factors in Table 2 (varied from 2.2 to 3.0), and even larger for WHO-5 and PROMIS-physical: the difference between participating GPs and non-participating GPs on the WHO-5 scale increased with 0.9 points to 2.6 after controlling for case-mix (*p* < 0.01), while the difference on the PROMIS-physical scale increased by 1.1 points to 2.6 (*p* = 0.002).

**Table 3:** Comparisons of chronic patients’ experiences- and outcomes ^a^ between participating and non-participating GPs

	Patients from participating GPs (*n* = 747)	Patients from non-participating GPs (*n* = 2,612)	*P* value
*Patient experiences, P3CEQ:*			
Index (10 items)^b^	58.2 (24.8)	55.7 (25.4)	0.017
Items:^c^			
Patients compelled to repeat information	74.3 (28.4)	70.4 (29.4)	0.002
Care joined up	73.8 (23.5)	70.8 (24.8)	0.005
Support to self-manage	60.4 (32.2)	58.9 (32.6)	0.370
Information to self-manage	58.2 (33.8)	57.7 (34.1)	0.777
Confidence to self-manage	51.9 (25.7)	51.1 (26.6)	0.487
Discuss what’s important to you	56.0 (29.0)	55.1 (29.5)	0.471
Involved in decisions	72.5 (28.3)	70.7 (27.9)	0.140
Considered “whole person”	79.3 (26.0)	77.9 (27.1)	0.236
*Overall evaluation of GP practice last 12 months* (item)	71.9 (23.1)	69.0 (24.3)	0.004
*WHO-5*:			
Scale (5 items)	65.3 (19.0)	63.6 (20.5)	0.039
Items:			
Cheerful and in good spirits	69.8 (20.3)	67.3 (21.4)	0.005
Calm and relaxed	70.3 (20.5)	68.1 (22.3)	0.015
Active and vigorous	58.4 (26.3)	57.3 (27.3)	0.332
Daily life filled with interesting things	67.3 (20.4)	65.3 (23.1)	0.028
Woke up fresh and rested	60.6 (27.1)	59.9 (27.8)	0.495
*PROMIS-physical:*			
Scale (2 items)	63.4 (20.5)	61.9 (21.6)	0.083
Items:			
Overall physical health	44.9 (22.2)	42.5 (23.0)	0.010
Carry out everyday physical activities	82.1 (24.2)	81.4 (25.4)	0.519
*PROMIS-mental*:			
Scale (2 items)	60.5 (20.6)	58.2 (22.2)	0.013
Items:			
Overall mental health	63.0 (23.1)	60.2 (24.6)	0.005
Satisfaction with social activities and relationships	58.0 (22.8)	56.3 (24.4	0.080

^a^Scales, index and items transformed linearly to a 0-100 scale, where 100 is the best experience/outcome. SD in parenthesis

^b^The index is the average sum of ten items dichotomized, transformed into a 0-100 scale

^c^The two items with two response categories in the questionnaire are excluded from results for items (having a responsible health personnel; having a care plan)

## Discussion

None of the GP characteristics differed between participating and non-participating GPs, and patients from the two groups were only slightly different on a few background variables. Persons with chronic conditions from participating GPs reported somewhat better health care experiences, well-being and mental health than persons with chronic conditions from non-participating GPs.

The PaRIS survey has similarities both with the Commonwealth Fund international health policy survey and the EUROPEP study [3, 8], but even more with the QUALICOPC study (Quality and Costs of Primary Care in Europe), which focused on cross-country comparisons of primary care based on a multilevel design and patient-reported indicators [22]. Research related to methodological issues in the Commonwealth Fund international survey is scarce [23], and methodological research related to EUROPEP has mostly been limited to psychometric evaluations of the measurement instrument [24, 25]. The QUALICOPC study has documented recruitment procedures and participation rates for all participating countries [4], and published a study about the representativeness of the Canadian results [9]. However, the QUALICOPC study has not assessed possible GP selection bias on the representativeness of the patient data in any of the participating countries. Thus, we are not aware of other studies on this topic in the context of a large-scale international health care quality survey.

All in all, our study indicates a modest selection bias for the PaRIS survey when only including patients from participating GPs, resulting in somewhat overestimated PREMs and PROMs values at the national level (1.5–2.9 points on a 0-100 scale). This bias will not be corrected by non-response weighting of the GP sample, as the GP and GP practice characteristics of participating and non-participating GPs were similar. Weighting the patient sample is not a solution either, as the differences were the same or larger after adjusting for patient characteristics. The most likely explanation is that GPs who chose to participate have more interest in chronic care and/or better chronic care performance than the average GP and that it would also be linked with differences in the relevant experiences and outcomes of care. This is consistent with the leverage-saliency theory [7], with the saliency of chronic care in the invitation to GPs producing this effect. Previous research has shown that GPs are more likely to respond if they perceive the survey as highly interesting [26].

The scarcity of studies on this topic means that more research is needed within and across countries to assess and adjust for potential selection bias. The Norwegian part of the study has legal approvals and access to a national register with patients nested within providers (GPs), facilitating this kind of research. The Norwegian infrastructure is well suited for multilevel surveys like the PaRIS survey and will be used to assess and monitor selection bias in further iterations of the survey. An explanation for the scarcity of research on selection bias could be that many countries face legal or technical challenges when conducting multilevel surveys like the PaRIS survey. Other countries should conduct similar research to secure fair and valid cross-country comparisons. If there are juridical or technical difficulties in including patients from non-responding providers, modified approaches could be used. One possible approach would be to use late provider participants as a proxy for non-respondents, requiring only a standardized invitation date and dates for provider responses/participation. In a so-called wave-analysis, PREMs and PROMs for late vs. early providers could be compared, the former being most similar to non-respondents because they are closest on the continuum of resistance to participate in the survey [27]. Another approach could be to conduct a similar and parallel survey in a representative patient sample, but without the connection to providers. This would demand the same questionnaire, the same population and the same data collection procedures as in the main survey. Access to a sampling frame with the same population could be difficult, especially the requirement of having at least one consultation with a primary care provider the last six months. However, this could be included as a question in the questionnaire, facilitating the creation of a comparable sample a posteriori and comparisons with results from the standard PaRIS approach. A third approach could be follow-up studies of provider non-respondents, probing the reasons for refusal [26] or using other data collection modes with the same provider questionnaire [23] or preferably a shorter questionnaire [28]. Provider responses from previous non-respondents could then be compared with provider respondents, especially to assess potential differences in self-reported structures, processes and systems for chronic health care. Feasibility and/or costs might prohibit many countries from conducting these alternative approaches, so it would nevertheless be important to exploit knowledge from other countries having the same opportunity as Norway to assess selection bias (e.g., France and Wales). The Norwegian field trial is the first study on selection bias in the PaRIS survey, which later should be interpreted together with evidence from other countries when assessing the potential effect of selection bias on national representativeness and cross-country comparisons.

### Limitations

One limitation of the current study is that it was based on the questionnaire and data collection procedures in the PaRIS field trial. Given changes in the questionnaire, samples and data collection procedures before the main survey, field trial findings might not be generalizable to the main survey. The proposed changes from the field trial to the main survey, however are small, the largest being the sample sizes where the number of providers (and thus the total number of patients) is much higher than in the field trial. In theory, the changes are unrelated to selection bias, but we will assess selection bias following the main survey to evaluate consistency over time. Another limitation is that we lack GP level survey measurement for comparison, obviously a result of GP survey non-response constituting non-participating GPs in our study. A third limitation is that the study was restricted to one participating country with uncertain generalizability to the other countries. We underline the importance of more research on selection bias in the PaRIS survey, which also could be relevant for multilevel research studies and surveys on other topics.

### Implications

The first implication of our findings is the need for an increased awareness of and research on selection bias as a potential source of bias in research and surveys with multilevel design. The Norwegian field trial somewhat overestimated PREMs and PROMs, which without corrections beyond non-response weighting and case-mix adjustment could influence the validity of comparisons with other countries. In addition, more initiatives to mitigate selection bias are needed. A systematic review of the literature showed that survey topic in digital surveys is one of the most important factors for obtaining a survey response, with the odds of response almost doubled if the invited person finds the topic interesting [29]. To avoid only recruiting GPs/providers being especially interested in chronic care, the invitation letter and reminders could vary the saliency of different attributes in the survey requests. E.g., the multiple reminder strategy in the Norwegian PaRIS survey could be used to tailor the content of reminders so that the first reminder focuses on chronic care, the second reminder on the monetary incentive and the third on a combination of incentive and short questionnaire. In accordance with the leverage-saliency theory [7], the importance of these attributes will vary between different persons or sub-groups and by varying the visibility of these attributes in the reminders we might avoid recruiting only GPs with a special interest in chronic care. The goal is to end up with a representative sample of providers, where reasons for participation are unrelated to the study topic of chronic care performance. Whether or not these initiatives actually reduce selection bias should be assessed in future studies. As always, initiatives to secure a high response rate are warranted [30], which also could reduce selection bias if late respondents are less interested/have poorer chronic care performance than early respondents.

If the selection bias estimates from our study were fixed across time and countries the only consequence would be a slight general overestimation of PREMs and PROMs, with no consequences for cross-country comparisons. However, the current lack of evidence across countries means that we cannot conclude about a fixed effect, which creates uncertainty when comparing results across countries. Furthermore, selection bias is only one of potential many sources of survey error, and a new, complex, and high-stake international survey like the PaRIS survey should document and address as many of these as possible to secure valid comparisons. Even though selection bias in our study was only modest, the combined effect of this and other error sources could potentially be large. To aid interpretations and use in health policy, the first PaRIS survey flagship report should include a chapter about methodological issues including research and considerations related to selection bias.

## Conclusions

Persons with chronic conditions from participating GPs reported better experiences, well-being and mental health than persons with chronic conditions from non-participating GPs in the PaRIS survey. Although the differences were modest, the Norwegian field trial indicates somewhat overestimated patient-reported experiences and -outcomes at the aggregated level and the need for more research within and across countries to identify and adjust for this potential bias.

## Supplementary Information

Supplementary Material 1.

Supplementary Material 2.

## Data Availability

The dataset is available from the corresponding author on reasonable request.

## Abbreviations

PaRIS: patient-reported indicator survey
PREMs: patient-reported experience measures
PROMs: patient-reported outcome measures
GP: general practitioner
PCP: primary care provider
DPIA: data protection impact assessment
P3CEQ: patient-centred coordinated care experience questionnaire
WHO-5: WHO well-being index, 5 items
EUROPEP: European task force on patient evaluations of general practice care
QUALICOPC: quality and costs of primary care in Europe

## References

[1] Valderas JM, Gangannagaripalli J, Nolte E, Boyd CM, Roland M, Sarria-Santamera A, Jones E, et al. Quality of care assessment for people with multimorbidity. J Intern Med. 2019;285:289–300. 30719790 10.1111/joim.12881

[2] de Boer D, van den Berg M, Ballester M, Bloemeke J, Boerma W, de Bienassis K, et al. Assessing the outcomes and experiences of care from the perspective of people living with chronic conditions, to support countries in developing people-centred policies and practices: study protocol of the International Survey of people living with chronic conditions (PaRIS survey). BMJ Open. 2022;12:e061424. 36123114 10.1136/bmjopen-2022-061424 PMC9486339

[3] Osborn R, Doty MM, Moulds D, Sarnak DO, Shah A. Older americans were Sicker and Faced more financial barriers to Health Care Than counterparts in other Countries. Health Aff (Millwood). 2017;36:2123–32. 29140737 10.1377/hlthaff.2017.1048

[4] Groenewegen PP, Greß S, Schäfer W. General practitioners’ participation in a large, Multicountry Combined General Practitioner-Patient Survey: recruitment procedures and participation rate. Int J Family Med. 2016;2016:4929432. 27047689 10.1155/2016/4929432 PMC4800081

[5] Norman RM, Bjertnæs ØA, Danielsen K, Holmboe O. Pasienterfaringer med fastlegen og fastlegekontoret i 2021/2022. [Patient experience with the general practitioner and the general practitioner office in 2021/2022.] PasOpp-rapport 2022. Oslo: Folkehelseinstituttet; 2022. p. 566.

[6] Groves RM, Fowler FJ, Couper MP, Lepkowski JM, Singer E, Tourangeau R. Survey Methodology. Hoboken: Wiley; 2009.

[7] Groves RM, Singer E, Corning A. Leverage-saliency theory of survey participation: description and an illustration. Public Opin Q. 2000;64:299–308. 11114270 10.1086/317990

[8] Grol R, Wensing M, Mainz J, Ferreira P, Hearnshaw H, Hjortdahl P, et al. Patients’ priorities with respect to general practice care: an international comparison. European Task Force on patient evaluations of General Practice (EUROPEP). Fam Pract. 1999;16:4–11. 10321388 10.1093/fampra/16.1.4

[9] Li A, Cronin S, Bai YQ, Walker K, Ammi M, Hogg W, et al. Assessing the representativeness of physician and patient respondents to a primary care survey using administrative data. BMC Fam Pract. 2018;19:77. 29848292 10.1186/s12875-018-0767-9 PMC5977493

[10] Eide TB, Straand J, Melbye H, Rortveit G, Hetlevik I, Rosvold EO. Patient experiences and the association with organizational factors in general practice: results from the Norwegian part of the international, multi-centre, cross-sectional QUALICOPC study. BMC Health Serv Res. 2016;16:428. 27553244 10.1186/s12913-016-1684-z PMC4995793

[11] Saunes IS, Karanikolos M, Sagan A, Norway. Health system review. Health Syst Transition. 2020;22(1):i–163. 32863241

[12] Eide TB, Straand J, Björkelund C, Kosunen E, Thorgeirsson O, Vedsted P, et al. Differences in medical services in nordic general practice: a comparative survey from the QUALICOPC study. Scand J Prim Health Care. 2017;35:1–10. 10.1080/02813432.2017.1358856 28768442

[13] Porter I, Rijken M, Groene O, Suñol R, Williams R, van den Berg M, et al. The International Survey of people living with chronic conditions (PaRIS survey): development of the patient questionnaire. Qual Life Res. 2021;30(Suppl 1):S42.

[14] Porter I, Rijken M, Groene O, Suñol R, Williams R, van den Berg M, et al. The International Survey of people living with chronic conditions (PaRIS survey): development of the conceptual framework. Qual Life Res. 2021;30(Suppl 1):S48.

[15] Valderas JM, Porter I, Martin-Delgado J, Rijken M, de Jong J, Groene O, et al. Development of the Patient-Reported Indicator Surveys (PaRIS) conceptual framework to monitor and improve the performance of primary care for people living with chronic conditions. BMJ Qual Saf. 2024;5:bmjqs-2024. 10.1136/bmjqs-2024-017301 39174334

[16] Lloyd H, Fosh B, Whalley B, Byng R, Close J. Validation of the person-centred coordinated care experience questionnaire (P3CEQ). Int J Qual Health Care. 2019;31:506–12. 30508089 10.1093/intqhc/mzy212 PMC6839368

[17] Sollid MIV, Slaaen M, Danielsen S, Kirkevold Ø. Psychometric properties of the person-centred coordinated care experience questionnaire (P3CEQ) in a Norwegian radiotherapy setting. Int J Qual Health Care. 2022;34:mzac067. 36004618 10.1093/intqhc/mzac067 PMC9475430

[18] Sizmur S, Graham C, Bos N. Psychometric evaluation of patient-reported experience measures: is it valid? Int J Qual Health Care. 2020;32:219–20. 32103271 10.1093/intqhc/mzaa006

[19] Crow R, Gage H, Hampson S, Hart J, Kimber A, Storey L, Thomas H. The measurement of satisfaction with healthcare: implications for practice from a systematic review of the literature. Health Technol Assess. 2002;6:1–244. 12925269 10.3310/hta6320

[20] Topp CW, Østergaard SD, Søndergaard S, Bech P. The WHO-5 well-being index: a systematic review of the literature. Psychother Psychosom. 2015;84:167–76. 25831962 10.1159/000376585

[21] Hays RD, Schalet BD, Spritzer KL, Cella D. Two-item PROMIS^®^ global physical and mental health scales. J Patient Rep Outcomes. 2017;1:2. 29757325 10.1186/s41687-017-0003-8 PMC5934936

[22] Schäfer WL, Boerma WG, Kringos DS, De Maeseneer J, Gress S, Heinemann S, Rotar-Pavlic D, et al. QUALICOPC, a multi-country study evaluating quality, costs and equity in primary care. BMC Fam Pract. 2011;12:115. 22014310 10.1186/1471-2296-12-115 PMC3206822

[23] Bjertnaes OA, Iversen HH, Bukholm G. International health policy survey in 11 countries: assessment of non-response bias in the Norwegian sample. BMC Health Serv Res. 2010;10:38. 20146819 10.1186/1472-6963-10-38 PMC2833149

[24] Evans RG, Edwards A, Evans S, Elwyn B, Elwyn G. Assessing the practising physician using patient surveys: a systematic review of instruments and feedback methods. Fam Pract. 2007;24:117–27. 17264071 10.1093/fampra/cml072

[25] Godillot C, Jendoubi F, Konstantinou MP, Poncet M, Bergeron A, Gallini A, et al. How to assess patient satisfaction regarding physician interaction: a systematic review. Dermatol Ther. 2021;34:e14702. 33368997 10.1111/dth.14702

[26] Kaner EF, Haighton CA, McAvoy BR. So much post, so busy with practice–so, no time!‘: a telephone survey of general practitioners’ reasons for not participating in postal questionnaire surveys. Br J Gen Pract. 1998;48:1067–9. 9624749 PMC1410021

[27] Halbesleben JR, Whitman MV. Evaluating survey quality in health services research: a decision framework for assessing nonresponse bias. Health Serv Res. 2013;48:913–30. 23046097 10.1111/1475-6773.12002 PMC3681236

[28] Garratt AM, Bjertnaes OA, Holmboe O, Hanssen-Bauer K. Parent experiences questionnaire for outpatient child and adolescent mental health services (PEQ-CAMHS outpatients): reliability and validity following a national survey. Child Adolesc Psychiatry Ment Health. 2011;5:18. 21600010 10.1186/1753-2000-5-18 PMC3120777

[29] Edwards PJ, Roberts I, Clarke MJ, DiGuiseppi C, Woolf B, Perkins C. Methods to increase response to postal and electronic questionnaires. Cochrane Database Syst Rev. 2023;11(11):MR000008. 38032037 10.1002/14651858.MR000008.pub5 PMC10687884

[30] Pit SW, Vo T, Pyakurel S. The effectiveness of recruitment strategies on general practitioner’s survey response rates - a systematic review. BMC Med Res Methodol. 2014;14:76. 24906492 10.1186/1471-2288-14-76 PMC4059731

