# Supplementary material for: International survey of people living with chronic conditions (PaRIS survey): effects of general practitioner non-participation on the representativeness of the Norwegian patient data
Source: BMC Health Serv Res. 2024 Oct 18;24:1257. doi: 10.1186/s12913-024-11751-0 (PMC11487967; doi:10.1186/s12913-024-11751-0)
Supplement: Supplementary file 1 — Supplementary Material 1. [file 12913_2024_11751_MOESM1_ESM.pdf]

PaRIS
Patient Reported Indicator Surveys

PaRIS Patient Questionnaire (PaRIS-PQ)

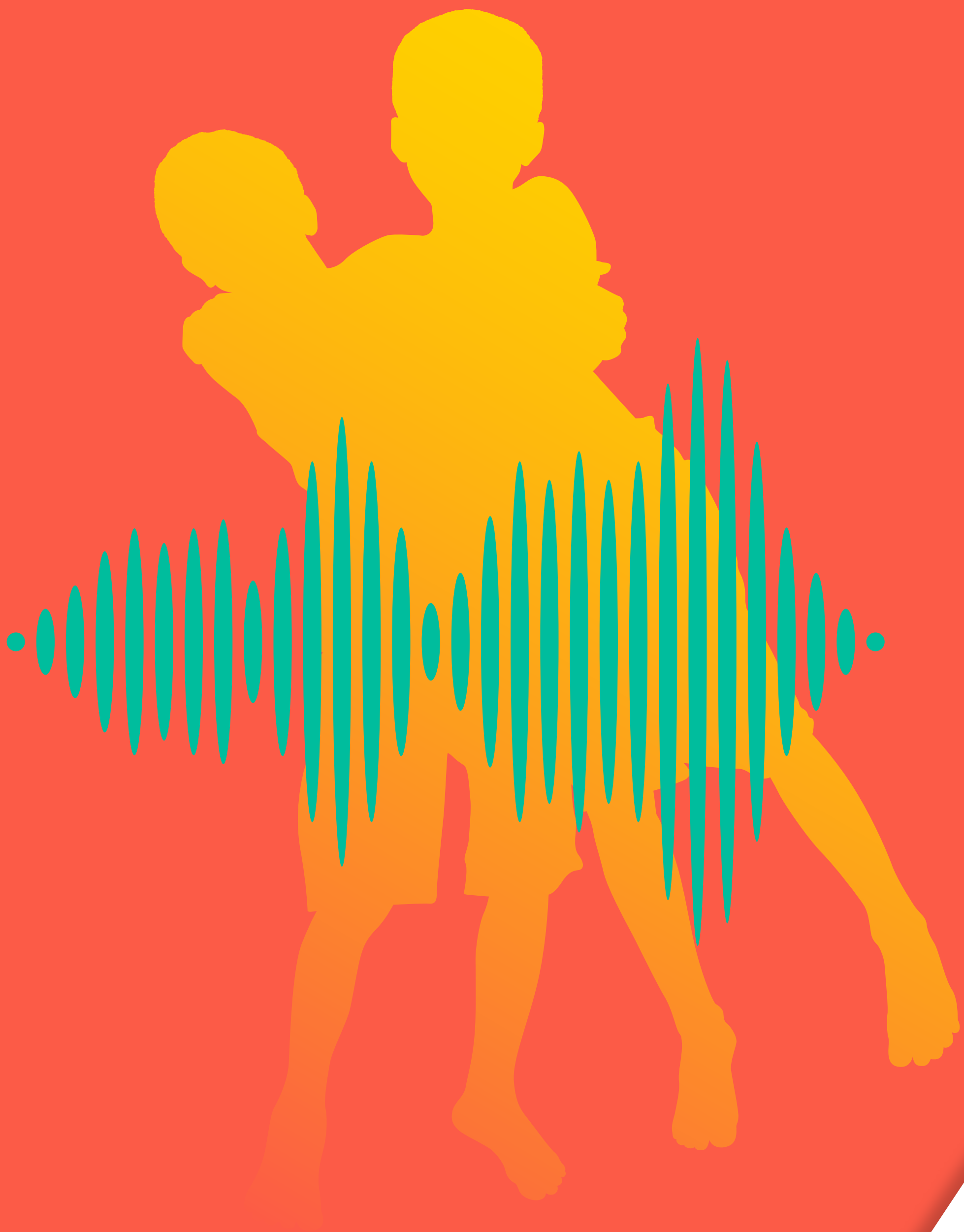

OECD

OECD PaRIS Patient Questionnaire (PaRIS-PQ)

Version for scripting online survey
2024

© OECD 2024 The use of this work, whether digital or print, is governed by the Terms and Conditions to be found at https://www.oecd.org/termsandconditions.

Table of contents

Background 3
OECD PaRIS-PQ for scripting 4

Annex A. Source questionnaires of the PaRIS-PQ 41

Background

The OECD's Patient-Reported Indicator Surveys (PaRIS) initiative aims to evaluate healthcare outcomes and patient experiences internationally, with a focus on primary care performance. The primary tool used in this initiative is the PaRIS Patient Questionnaire (PaRIS-PQ), designed to assess the experiences and outcomes of patients aged 45 years and older managed in primary care. Alongside patient-reported outcomes and experience measures (PROMs and PREMs), the PaRIS-PQ includes items about sociodemographic factors, health behaviours, and healthcare capabilities. To offer further context and insights into patient-reported experiences and outcomes, a complementary questionnaire called the PaRIS Primary Care Practice Questionnaire (PaRIS-PCPQ) is employed to evaluate practice characteristics and the care delivered, particularly focusing on chronic condition management.

The development of the PaRIS-PQ involved close collaboration with policymakers, patients, and primary care providers. It is grounded in the PaRIS conceptual framework, developed through a rigorous process involving literature review and a modified Delphi process. The questionnaire underwent translation into the languages of participating countries, cross-national cognitive testing, and pilot testing in a field trial.

This document presents the English source version of the PaRIS-PQ for scripting an online survey, used for data collection between 2022 and 2023. Additionally, it includes the sources for the items incorporated into the PaRIS-PQ.

The PaRIS Initiative (Patient-Reported Indicator Surveys) of the OECD aims to develop and implement internationally comparable patient-reported indicators, more specifically patient-reported outcome measures (PROMs) and patient-reported experience measures (PREMs).

The OECD developed the PaRIS survey instruments with an international consortium of Nivel, Ipsos, University of Exeter, Optimedis AG and Avedis Donabedian Research Institute.

OECD PaRIS-PQ for scripting

Note to the reader: The PaRIS PQ was administered mainly online, but also on paper and by telephone depending on the best suited mode for the country. The layout of this document uses the PaRIS PQ version for scripting the online survey, rather than the paper-based version. Please note following elements for clarification:

- ASK ALL: item is applicable to all respondents
- ASK IF: applicability of the item is conditional on another item
- (SINGLE): items where respondents can give one of the response options
- (MULTI): items where respondents may tick multiple response options
- (LOOP): items consisting of multiple questions, each with the same answering categories

This survey is being carried out by <<insert national relevant organization in each country>> in collaboration with the Organisation for Economic Cooperation and Development (OECD). <<insert country>> is taking part in an international survey to improve health services locally and nationally.

The survey asks questions about your experience of care and treatment by your <<primary care provider>>. *Primary care services are usually the first point of contact with the health service for a wide range of new and long-term health problems. They are frequently delivered by teams of health care professionals, usually coordinated by a family doctor or a general practitioner working in a primary care centre. In your case this may be also another member of the primary care team, such as a nurse, or another specialist doctor.*

In addition, there are some questions about you and your health. Your answers are important and will help policy makers and health care providers improve health care based on patients' needs.

The survey should take around 25 minutes, depending on the answers you give. If you cannot complete the survey in one session and would like to come back and finish later, just close the window and your responses will be saved. When you are ready, you can return to the same point.

Taking part in this survey is voluntary and your answers will be treated in confidence. If you do not wish to answer a question, please click the >> button twice to move on.

For more information about this survey and how we will use your data, please click on the link below to view the privacy policy.

If you have any queries about the questionnaire, please call our helpline number on [LOCAL NUMBER] or email [LOCAL EMAIL].

CONSENT (SINGLE CODE)

Do you agree to take part in the survey?

Yes CONTINUE

No CLOSE

SECTION 1 “Your Health” TIMING START

ASK ALL
NEW PAGE

Your health

The first few questions are about your health.

Questions	Source
Q_AGE (SINGLE)<br>**How old are you?**<br><br>1 – 44 years old or younger<br>2 – 45-49 years old<br>3 – 50-54 years old<br>4 – 55-59 years old<br>5 – 60-64 years old<br>6 – 65-69 years old<br>7 – 70-74 years old<br>8 – 75-79 years old<br>9 – 80-84 years old<br>10 – 85 years or older<br>97 – Prefer not to say	Modified from OECD PISA-D Main Study Teacher Questionnaire IS

IF Q_AGE = 1 PARTICIPANTS ARE INELIGIBLE AND SHOULD NOT CONTINUE

ASK ALL
NEW PAGE

Questions	Source
Q_GLOBAL (LOOP)<br>GLOBAL01 – **In general, would you say your health is:**<br>GLOBAL02 – **In general, would you say your quality of life is:**<br>GLOBAL03 – **In general, how would you rate your physical health?**<br>GLOBAL04 – **In general, how would you rate your mental health, including your mood and your ability to think?**<br>GLOBAL05 – **In general, how would you rate your satisfaction with your social activities and relationships?**<br>GLOBAL9R – **In general, please rate how well you carry out your usual social activities and roles. (This includes activities at home, at work and in your community, and responsibilities as a parent, child, spouse, employee, friend, etc.)**<br><br>Responses (SINGLE)<br>1 – Excellent<br>2 – Very good<br>3 – Good<br>4 – Fair<br>5 – Poor	PROMIS® Scale v1.2 – Global Health

ASK ALL
NEW PAGE

**Questions**	**Source**
Q_GLOBAL06 (SINGLE)<br>**To what extent are you able to carry out your everyday physical activities such as walking, climbing stairs, carrying groceries, or moving a chair?**<br>1 Completely<br>2 Mostly<br>3 Moderately<br>4 A little<br>5 Not at all	PROMIS® Scale v1.2 – Global Health

ASK ALL
NEW PAGE
The next few questions are about any specific symptoms you may have recently experienced.

ASK ALL
NEW PAGE

**Questions**	**Source**
Q_PROMISDYSCB001 (SINGLE)<br>**In the <u>past 7 days</u>**<br><br>**I have been short of breath**<br>1 Not at all<br>2 A little bit<br>3 Somewhat<br>4 Quite a bit<br>5 Very much	PROMIS Item Bank v. 1.0 – Dyspnea

ASK ALL
NEW PAGE

**Questions**	**Source**
Q_GLOBAL08R (SINGLE)<br>**In the <u>past 7 days</u>**<br><br>**How would you rate your fatigue on average?**<br>1 None<br>2 Mild<br>3 Moderate<br>4 Severe<br>5 Very severe	PROMIS® Scale v1.2 – Global Health

ASK ALL
NEW PAGE

Questions	Source
Q_PROMISPAIN (SINGLE)<br>**In the past 7 days**<br><br>**How much did pain interfere with your day-to-day activities?**<br>1 Not at all<br>2 A little bit<br>3 Somewhat<br>4 Quite a bit<br>5 Very much	PROMIS Adult Short Form v1.0 - Pain Interference

ASK ALL
NEW PAGE

Questions	Source
Q_GLOBAL07R (SINGLE)<br>**In the past 7 days**<br><br>**How would you rate your pain on average?**<br>0-No pain<br>1<br>2<br>3<br>4<br>5<br>6<br>7<br>8<br>9<br>10-Worst imaginable pain	PROMIS® Scale v1.2 – Global Health

ASK ALL
NEW PAGE

Questions	Source
Q_ GLOBAL10R (SINGLE)<br>**In the past 7 days**<br><br>**How often have you been bothered by emotional problems such as feeling anxious, depressed or irritable?**<br>1 Never<br>2 Rarely<br>3 Sometimes<br>4 Often<br>5 Always	PROMIS® Scale v1.2 – Global Health

ASK ALL
NEW PAGE

<table>
<tr><th>Questions</th><th>Source</th></tr>
<tr><td>
(REPEAT ON EACH SCREEN) For the next few questions, please think about how you have been feeling over <u>the last two weeks</u>.

QWHO5 (LOOP)
<table>
<tr><td>A</td><td>I have felt cheerful and in good spirits</td></tr>
<tr><td>B</td><td>I have felt calm and relaxed</td></tr>
<tr><td>C</td><td>I have felt active and vigorous</td></tr>
<tr><td>D</td><td>My daily life has been filled with things that interest me</td></tr>
<tr><td>E</td><td>I woke up feeling fresh and rested</td></tr>
</table>

Responses (SINGLE)
<table>
<tr><td>1</td><td>All of the time</td></tr>
<tr><td>2</td><td>Most of the time</td></tr>
<tr><td>3</td><td>More than half of the time</td></tr>
<tr><td>4</td><td>Less than half of the time</td></tr>
<tr><td>5</td><td>Some of the time</td></tr>
<tr><td>6</td><td>At no time</td></tr>
</table>
</td><td>WHO Well-being Index (WHO-5)</td></tr>
</table>

SECTION 1 'Your health' TIMING END

SECTION 2 ‘Managing your health and health care’ TIMING START

ASK ALL
NEW PAGE

Managing your health and health care

The next few questions ask about your recent physical activity, diet and use of tobacco and alcohol and the support you may have received from health care professionals (doctors, nurses, and other health care professionals). Please respond in relation to your experience of care and treatment by your <<primary care provider>>. Primary care services are usually the first point of contact with the health service for a wide range of new and long-term health problems.

ASK ALL
NEW PAGE (SAME SCREEN)

Questions	Source
Q_EXERCISE (SINGLE)<br>**In the past week, on how many days did you do at least 30 minutes of either vigorous or moderate activity (such as running, swimming, riding a bike, brisk walking, or dancing)? Vigorous physical activities typically make you breathe much harder than normal, whereas moderate activities make you breathe somewhat harder than normal.**	Modified from IPAQ-SF

1	Every day
2	5 to 6 days
3	3 to 4 days
4	1 to 2 days
5	I did not do any vigorous or moderate activity in the past week
6	Not sure

ASK ALL
NEW PAGE

Questions	Source
Q_ADVPHYSACT (SINGLE)<br>**In the past 12 months, has any health care professional talked with you about your physical activity?**	Modified from CWF2017IHP

1	Yes
2	No
3	Not sure

ASK ALL
NEW PAGE

**Questions**	**Source**
Q_EHISFR (SINGLE)<br>**How often do you eat fruit, excluding juice squeezed from fresh fruit or made from concentrate?**	Modified from European Health Interview 2020 Edition

1	More than once a day
2	Once a day
3	4 to 6 days a week
4	1 to 3 days a week
5	Less than once a week
6	Never

ASK ALL
NEW PAGE

**Questions**	**Source**
Q_EHISVG (SINGLE)<br>**How often do you eat vegetables or salad, excluding potatoes and fresh juice or juice made from concentrate?**	Modified from European Health Interview 2020 Edition

1	More than once a day
2	Once a day
3	4 to 6 days a week
4	1 to 3 days a week
5	Less than once a week
6	Never

ASK ALL
NEW PAGE

**Questions**	**Source**
Q_ ADVDIET (SINGLE)<br>**In the past 12 months, has any health care professional talked with you about healthy eating?**	Modified from CWF2017IHP

1	Yes
2	No
3	Not sure

ASK ALL
NEW PAGE

Questions	Source
Q_EHISSK01 (SINGLE)<br>**Do you smoke any tobacco products (excluding electronic cigarettes or similar electronic devices)?**<br>1 — Yes, daily<br>2 — Yes, occasionally<br>3 — Not at all	European Health Interview 2020 Edition

ASK IF Q_EHISSK01 = 3
NEW PAGE

Questions	Source
Q_EHISSK04 (SINGLE)<br>**Have you ever smoked tobacco products (excluding electronic cigarettes or similar electronic devices) daily, or almost daily, for at least one year?**<br>1 — Yes<br>2 — No	Modified from European Health Interview 2020 Edition

ASK IF Q_EHISSK01 = 1 or 2 OR Q_EHISSK04=1
NEW PAGE

Questions	Source
Q_CWF2017IHP (SINGLE)<br>**In the past 12 months, has any health care professional talked with you about the health risks of smoking or using tobacco and ways to quit?**<br>1 — Yes<br>2 — No<br>3 — Not sure	Modified from CWF2017IHP

ASK ALL

NEW PAGE

Questions	Source
Q_EHIS (SINGLE)<br>**In the past 12 months, how often have you had an alcoholic drink of any kind (beer, wine, cider, spirits, cocktails, premixes, liquor, homemade alcohol …)?**	European Health Interview 2020 Edition

**1**	Every day or almost
**2**	5-6 days a week
**3**	3-4 days a week
**4**	1-2 days a week
**5**	2-3 days in a month
**6**	Once a month
**7**	Less than once a month
**8**	Not in the past 12 months, as I no longer drink alcohol
**9**	Never, or only a few sips or tries, in my whole life

ASK IF Q_EHIS = 1 TO 8

NEW PAGE

Questions	Source
Q_CWF2017IHP (SINGLE)<br>**In the past 12 months, has any health care professional talked with you about alcohol use?**	Modified from CWF2017IHP

1	Yes
2	No
3	Not sure

SECTION 2 ‘Managing your health and health care’ TIMING END

SECTION 3 ‘PN and CONFIDENT’ TIMING START

ASK ALL

NEW PAGE

Q_PN (show each question on separate screens)

(Put this text on separate screen). The next few questions are about how you and the health care professionals that you usually see support your health and health care. Please respond in relation to your experience of care and treatment by your <<primary care provider>>.

	**Questions**	**Source**
PN1	**I rely on health care professionals to tell me everything I need to know to manage my health**	Modified from Porter-Novelli Consumer Preferences Scale
PN2	**Most health issues are too complex for me to understand**	Porter-Novelli Consumer Preferences Scale
PN3	**I actively try to prevent diseases and illnesses**	Porter-Novelli Consumer Preferences Scale
PN4	**I leave it to health care professionals to make the right decisions about my health**	Modified from Porter-Novelli Consumer Preferences Scale
PN5	**It is important to me to be informed about health issues**	Porter-Novelli Consumer Preferences Scale
PN6	**I need to know about health issues so I can keep myself and my family healthy**	Porter-Novelli Consumer Preferences Scale
PN7	**I have difficulty understanding a lot of the health information that I read**	Porter-Novelli Consumer Preferences Scale
PN8	**My health care professionals and I work together to manage my health**	Modified from Porter-Novelli Consumer Preferences Scale
PN9	**When I read or hear something that is relevant to my health care, I bring it up with my health care professionals**	Modified from Porter-Novelli Consumer Preferences Scale
PN10	**I try to understand my personal health risks**	Porter-Novelli Consumer Preferences Scale
PREOS2m	**When I think something is wrong with my health care, I raise my concerns with my health care professionals**	Modified from PREOS-PC-6

Responses (SINGLE)

1	Strongly disagree
2	Disagree
3	Neither agree nor disagree
4	Agree
5	Strongly agree

ASK ALL
NEW PAGE
Q_CONFIDENT (LOOP)

	**Questions**	**Source**
P3CEQ10	**How confident are you that you can manage your own health and wellbeing?**	Modified from Person-Centred Coordinated Care Experience Questionnaire (P3CEQ)
MPBSPAINSTRC	**How confident are you that you can follow instructions from health care professionals about how you should care for yourself at home?**	Modified from Medicare Patient Engagement Questions
MPBSPAMEDREC	**How confident are you that you can follow instructions from health care professionals about how to change your habits or lifestyle?**	Modified from Medicare Patient Engagement Questions
MPBSPANECESS	**How confident are you that you can identify when it is necessary for you to get medical care?**	Modified from Medicare Patient Engagement Questions
MPBSPASIDEFX	**How confident are you that you can identify when you are having side effects from your medications?**	Modified from Medicare Patient Engagement Questions
eHEALS	**How confident are you in using information from the Internet to make health decisions?**	Modified from eHealth Literacy Scale (eHEALS)

Responses (SINGLE)

1	Very confident
2	Confident
3	Somewhat confident
4	Not confident at all
5	Does not apply (MPBSPASIDEFX and eHEALS only)

SECTION 3 ‘PN and CONFIDENT’ TIMING END

SECTION 4 ‘Your experience of health care – part 1’ TIMING START

ASK ALL
NEW PAGE

Your experience of health care

Please respond in relation to your experience of care and treatment by your <<primary care provider>>.

ASK ALL
NEW PAGE
Q_CWF1130 (SINGLE)

Questions	Source
**Is there one single professional you usually go to for most of your health problems?**	Modified from CWF2016IHP

1	Yes, a doctor
2	Yes, another health care professional
3	No, there is no single health care professional I usually go for most of my health problems
4	Not sure

ASK IF Q_CWF1130 = 1
NEW PAGE
Q_ USUALDR1 (SINGLE)

Questions	Source
**Who is this doctor?**	OECD PaRIS

1	A doctor specialised in family medicine/general practice
2	Another specialist doctor
3	Not sure

ASK IF Q_CWF1130 = 1
NEW PAGE
Q_CWF1140 (SINGLE)

Questions	Source
**How long have you been seeing this doctor?**	Modified from CWF2016IHP

1	1 year or less
2	More than 1 year but no more than 3 years
3	More than 3 years but no more than 5 years
4	More than 5 years but no more than 10 years
5	More than 10 years
6	Not sure

ASK IF Q_CWF1130 = 1
NEW PAGE
Q_ USUALDR2 (SINGLE)

Questions	Source
**How many times have you seen this doctor in the past 12 months? This could be in person, on the phone, by video call or online messaging.**	OECD PaRIS

1	Not in the past 12 months
2	1 time
3	2 times
4	3 times
5	4 times
6	5 times or more
7	Not sure

ASK IF Q_CWF1130 = 2
NEW PAGE
Q_ USUALDR3 (SINGLE)

Questions	Source
**Who is this health care professional?**	OECD PaRIS

1	A doctor specialised in family medicine/general practice
2	Another specialist doctor
3	A nurse
7	Nurse practitioner
4	An allied health care professional, such as a physiotherapist, dietician, or podiatrist
5	Another health care professional
6	Not sure

ASK IF USUALDR3 = 1-3
NEW PAGE
Q_ USUALDR4 (SINGLE)

Questions	Source
**How long have you been seeing this health care professional?**	OECD PaRIS

1	1 year or less
2	More than 1 year but no more than 3 years
3	More than 3 years but no more than 5 years
4	More than 5 years but no more than 10 years
5	More than 10 years
6	Not sure

ASK IF USUALDR3 = 1-3

NEW PAGE

Q_ USUALDR5 (SINGLE)

Questions	Source
**How many times have you seen this health care professional in the past 12 months? This could be in person, on the phone, by video call or online messaging.**	OECD PaRIS

1	Not in the past 12 months
2	1 time
3	2 times
4	3 times
5	4 times
6	5 times or more
7	Not sure

SECTION 4 'Your experience of health care – part 1' TIMING END

SECTION 5 ‘Your experience of health care – part 2’ TIMING START

ASK ALL
NEW PAGE
Q_CHRONCOND (MULTI, OPEN)

Questions	Source
**Have you ever been told by a doctor that you have any of the following health conditions?**<br>Please select all the options that apply.	OECD PaRIS

1	High blood pressure
2	Cardiovascular or heart condition
3	Diabetes (type 1 or 2)
4	Arthritis or ongoing problem with back or joints
5	Breathing condition (e.g., asthma or COPD)
6	Alzheimer’s disease or other cause of dementia
7	Depression, anxiety or other mental health condition (e.g., bipolar disorder or schizophrenia) (ongoing)
8	Neurological condition (e.g., epilepsy or migraine)
9	Chronic kidney disease
10	Chronic liver disease
11	Cancer (diagnosis or treatment in the last 5 years)
12	Other long-term problem(s)
13	I have never been told by a doctor that I have any of these problems

ASK IF Q_CHRONCOND = 1 TO 12
NEW PAGE
The next few questions are about the care you may have received for these health conditions.

Please respond in relation to your experience of care and treatment by your << primary care provider>>.
Q_P3CEQ6 (SINGLE)

Questions	Source
**Do you have <u>a single professional</u> who takes responsibility for coordinating your care across the services that you use?**	Modified from Person-Centred Coordinated Care Experience Questionnaire (P3CEQ)

1	Yes
2	No
3	I do not receive care from more than one service
4	Not sure

ASK IF Q_CHRONCOND = 1 TO 12 AND Q_P3CEQ6 = 1
NEW PAGE
Q_ P3CEQ5m1 (SINGLE)

Questions	Source
**Is this the same professional that you see for most of your health problems?**<br><br>1 – Yes<br>2 – No<br>3 – Not sure	OECD PaRIS

ASK IF Q_CHRONCOND = 1 TO 12 AND Q_P3CEQ5m1 = 2 OR 3
NEW PAGE
Q_P3CEQ5m2 (SINGLE)

Questions	Source
**Who is this professional?**<br><br>1 – A doctor specialised in family medicine/general practice<br>2 – Another specialist doctor<br>3 – A nurse<br>7 – Nurse practitioner<br>4 – An allied health care professional, such as a physiotherapist, dietician, or podiatrist<br>5 – Another health care professional<br>6 – Not sure	OECD PaRIS

ASK IF Q_CHRONCOND = 1 TO 12
NEW PAGE
Q_P3CEQ5 (SINGLE)

Questions	Source
**Is your health care organised in a way that works for you?**<br><br>1 – Yes, definitely<br>2 – Yes, to some extent<br>3 – No, not really<br>4 – No, definitely not<br>5 – Not sure	Modified from Person-Centred Coordinated Care Experience Questionnaire (P3CEQ)

ASK IF Q_CHRONCOND = 1 TO 12
NEW PAGE
Q_ROUTINEF (SINGLE)

Questions		Source
**Are you offered regular follow-up for your health condition(s)? If you have more than one condition, please answer about the condition that you are seen for most regularly.**		OECD PaRIS
1	Yes, about every 3 months or more often	
2	Yes, about every 6 months	
3	Yes, about every 12 months	
4	Yes, over longer periods of time	
5	No	
6	Not sure	

ASK IF Q_CHRONCOND = 1 TO 12
NEW PAGE
Q_P3CE (LOOP)

Questions		Source
P3CEQ1	**Do you discuss with the health care professionals involved in your care what is most important for you in managing your own health and wellbeing?**	Modified from Person-Centred Coordinated Care Experience Questionnaire (P3CEQ)
P3CEQ2	**Are you involved as much as you want to be in decisions about your care?**	
P3CEQ3	**Are you considered as a 'whole person' rather than just a disease/condition in relation to your care?**	

Responses (SINGLE)

1	Yes, definitely
2	Yes, to some extent
3	No, not really
4	No, definitely not
5	Not sure

SECTION 5 'Your experience of health care – part 2' TIMING END

SECTION 6 ‘Your experience of health care – part 3’ TIMING START

ASK IF Q_CHRONCOND = 1 TO 12
NEW PAGE

Sometimes health professionals support patients in defining health goals. These are things that are important to you and you want help in achieving.

Q_PCMH4 (SINGLE)

Questions	Source
**How often does a health care professional talk to you about specific goals for your health?**	Modified from Patient-Centered Medical Home (PCMH)

1	Always
2	Often
3	Sometimes
4	Rarely
5	Never
6	Not sure

ASK IF Q_CHRONCOND = 1 TO 12 NEW PAGE
The next few questions are about <u>care plans</u>. A care plan (or plan of care) is a written agreement between you and your health care professionals detailing the type of care you receive over an extended period of time and how this care will be given, also including what you will do yourself. Not everybody has a care plan.
Please respond in relation to your experience of care and treatment by your <<primary care provider>>.

ASK IF Q_CHRONCOND = 1 TO 12
NEW PAGE
Q_P3CEQ7a

Questions	Source
**Do you have a care plan that takes into account all your health and wellbeing needs?**	Person-Centred Coordinated Care Experience Questionnaire (P3CEQ)

1	Yes
2	No
3	Not sure

ASK IF Q_CHRONCOND = 1 TO 12 AND Q_P3CEQ7a = 1
NEW PAGE
Q_P3CEQ7b (SINGLE)

Questions	Source
**Is this care plan available to you?**	Modified from Person-Centred Coordinated Care Experience Questionnaire (P3CEQ)

1	Yes
2	No
3	Not sure

ASK IF Q_CHRONCOND = 1 TO 12 AND Q_P3CEQ7a = 1
NEW PAGE
P3CEQ7c (SINGLE)

Questions	Source
**To what extent have you found your care plan useful for you to manage your health and wellbeing?**<br><br>1 Not at all<br>2 To some extent<br>3 To a large extent<br>4 Completely<br>5 Not sure	Modified from Person-Centred Coordinated Care Experience Questionnaire (P3CEQ)

ASK IF Q_CHRONCOND = 1 TO 12 AND Q_P3CEQ7a = 1
NEW PAGE
P3CEQ7d (SINGLE)

Questions	Source
**To what extent do all the professionals involved in your care appear to be following the same care plan?**<br><br>1 Not at all<br>2 To some extent<br>3 To a large extent<br>4 Completely<br>5 Not sure	Modified from Person-Centred Coordinated Care Experience Questionnaire (P3CEQ)

ASK IF Q_CHRONCOND = 1 TO 12
NEW PAGE
Q_P3CEQ8 (SINGLE)

Questions	Source
**Do you have enough support from your health care professionals to help you to manage your own health and wellbeing?**<br><br>1 I do not need support<br>2 I have had no support<br>3 I sometimes have enough support<br>4 I often have enough support<br>5 I always have enough support<br>6 Not relevant<br>7 Not sure	Modified from Person-Centred Coordinated Care Experience Questionnaire (P3CEQ)

ASK IF Q_CHRONCOND = 1 TO 12
NEW PAGE
P3CEQ9 (SINGLE)

**Questions**	**Source**
**To what extent do you receive useful information at the time you need it to help you manage your health and wellbeing?**<br><br>1 – I do not receive any information<br>2 – I sometimes receive enough information<br>3 – I often receive enough information<br>4 – I always receive enough information<br>5 – I receive too much information<br>6 – Not relevant<br>7 – Not sure	Modified from Person-Centred Coordinated Care Experience Questionnaire (P3CEQ)

ASK IF Q_CHRONCOND = 1 TO 12
NEW PAGE
Q_P3CEQ11b (SINGLE)

**Questions**	**Source**
**Do your health care professionals involve your family, friends and/or carers as much as you want them to be in decisions about your care?**<br><br>1 – No, definitely not<br>2 – No, not really<br>3 – Yes, to some extent<br>4 – Yes, definitely<br>5 – I do not want my family, friends or carers to be involved<br>6 – My family, friends or carers do not want to be involved<br>7 – Not applicable	Modified from Person-Centred Coordinated Care Experience Questionnaire (P3CEQ)

ASK IF Q_CHRONCOND = 1 TO 12
NEW PAGE
Q_MEDCWF1605 (SINGLE)

**Questions**	**Source**
**How many different medications as prescribed by a doctor or a nurse are you taking on a regular or ongoing basis?**<br><br>1 – No medication<br>2 – 1-2 medications<br>3 – 3-4 medications<br>4 – 5-9 medications<br>5 – 10 or more medications	Modified from CWF2016IHP

ASK IF Q_CHRONCOND = 1 TO 12 AND Q_MEDCWF1605 = 2 TO 5
NEW PAGE
Q_MEDCWF71110 (SINGLE)

Questions	Source
**In the past 12 months, has a health care professional reviewed with you all medications you take?**	Modified from CWF2017IHP

1	Yes
2	No
3	Not sure

SECTION 6 'Your experience of health care – part 3' TIMING END

SECTION 7 ‘Your experience of health care – part 4’ TIMING START

ASK ALL
NEW PAGE
The next few questions are about your experience with your <<primary care provider>> in the last 12 months.

Q_ P3CEQ4 (SINGLE)

Questions	Source
**Were there times when you had to repeat information that should have been in your care records?**<br><br>1 - Yes, definitely<br>2 - Yes, to some extent<br>3 - No, not really<br>4 - No, definitely not<br>5 - Not sure	Modified from Person-Centred Coordinated Care Experience Questionnaire (P3CEQ)

ASK ALL
NEW PAGE
Q_PREOS3 (SINGLE)
Sometimes patients experience an event or circumstance that could have resulted, or did result, in unnecessary harm to themselves, such as not getting an appointment when needed; receiving a wrong or delayed diagnosis or treatment; or experiencing problems with communications between health care professionals.

Questions	Source
**How often do you believe you have had any such event or circumstance with your primary care centre?**<br><br>1 - Always<br>2 - Often<br>3 - Sometimes<br>4 - Rarely<br>5 - Never<br>98 - Not applicable	Modified from PREOS-PC-6

ASK ALL
NEW PAGE
Q_PREOS1 (SINGLE)

Questions	Source
**How often did you feel that the health care professionals at your primary care centre encouraged you to talk about any concerns about your health care?**<br><br>1 - Always<br>2 - Often<br>3 - Sometimes<br>4 - Rarely<br>5 - Never<br>98 - Not applicable	Modified from PREOS-PC-6

ASK ALL
NEW PAGE
Q_OECDACC3 (SINGLE)

Questions	Source
**How often did you have a health problem but did not seek care because of difficulties in travelling to your primary care centre?**<br><br>1 Always<br>2 Often<br>3 Sometimes<br>4 Rarely<br>5 Never<br>98 Not applicable	Modified from OECD proposed Set of Questions on Patient Experiences with Ambulatory Care

ASK ALL
NEW PAGE
Q_ OECDACC46 (SINGLE)

Questions	Source
**How often did you have a health problem but did not seek care, or did not take a prescription medicine because of the cost?**<br><br>1 Always<br>2 Often<br>3 Sometimes<br>4 Rarely<br>5 Never<br>98 Not applicable	Modified from OECD proposed Set of Questions on Patient Experiences with Ambulatory Care

ASK ALL
NEW PAGE
The next questions are about the online services that your primary care centre may offer to patients like you, such as a website, smartphone app or video consultation

ASK ALL
NEW PAGE
Q_GPPS4 (SINGLE)

Questions	Source
**How easy is it for you to use your primary care centre's website to look for information or access services?**<br><br>1 Very easy<br>2 Fairly easy<br>3 Not very easy<br>4 Not at all easy<br>5 I haven't tried<br>6 My primary care centre does not have a website<br>7 Not sure	GP Patient Survey (GPPS)

ASK ALL
NEW PAGE
Q_GPPS5 (MULTI)

Questions	Source
**As far as you know, which of the following online services does your primary care centre offer?**<br>Please select all the options that apply.	Modified from GP Patient Survey (GPPS)

1	Booking appointments online
2	Ordering repeat prescriptions online
3	Accessing my medical records online
4	Video consultations
5	None of these
99	Don't know

ASK ALL
NEW PAGE
Q_GPPS6 (MULTIPLE)

Questions	Source
**Which of the following online services offered by your primary care centre have you used?**<br>Please select all the options that apply.	Modified from GP Patient Survey (GPPS)

1	Booking appointments online
2	Ordering repeat prescriptions online
3	Accessing my medical records online
4	Video consultations
5	None of these

ASK ALL
NEW PAGE
Q_CWF119 (SINGLE)

Questions	Source
**Overall, how do you rate the medical care that you have received in the past 12 months from your primary care centre?**	Modified from CWF2016IHP

1	Excellent
2	Very good
3	Good
4	Fair
5	Poor
6	Have not received medical care in the last 12 months
7	Not sure

SECTION 7 'Your experience of health care – part 4' TIMING END

SECTION 8 ‘Other health services’ TIMING START

ASK ALL
NEW PAGE

Other health services

The next few questions are about other health services you have used in the last 12 months.

ASK ALL
NEW PAGE
Q_ EMERG (SINGLE)

Questions	Source
**In the last 12 months, have you been to a hospital emergency department for your own medical care?**	Modified from CWF2017IHP

1	No
2	Yes, once
3	Yes, twice
4	Yes, 3 or more times
5	Not sure

ASK ALL
NEW PAGE
Q_CWF2016HOSP (SINGLE)

Questions	Source
**In the last 12 months, have you been in a hospital for one night or longer?**	Modified from CWF2016IHP

1	No
2	Yes, once
3	Yes, twice
4	Yes, 3 or more times
5	Not sure

SECTION 8 ‘Other health services’ TIMING END

SECTION 9 'Your last consultation' TIMING START

ASK ALL
NEW PAGE
Your last consultation **(SHOW ON SCREEN FOR ALL QS UP TO Q_OECDEXP5)**

The next questions are about the last time that you had a consultation with your <<primary care provider>> with a doctor, nurse or other health care professional to get care for yourself. This may have been with your main health care professional, or another health care professional, and includes consultations over the phone or in a doctor's office or clinic.

ASK ALL
NEW PAGE
LAST APP (country specific question to establish eligibility)

ASK ALL
NEW PAGE
Q_PURPOS (SINGLE)

Questions	Source
**What was the main purpose of this consultation?**	OECD PaRIS

1	First contact for a new problem
2	Follow-up of a new problem
3	Routine care for a long-term problem
4	Non-routine care for a long-term problem
5	General preventive care
6	Not sure

ASK ALL
NEW PAGE
Q_OECDACC7a (SINGLE)

Questions	Source
**Who did you have this last consultation with? If you saw more than one health care professional, please select the one you spent most time with on the day. Please select only one option.**	Modified from OECD proposed Set of Questions on Patient Experiences with Ambulatory Care

1	A doctor specialised in family medicine/general practice
2	Another specialist doctor
3	A nurse
7	Nurse practitioner
4	An allied health care professional, such as a physiotherapist, dietician, or podiatrist
5	Another health care professional
6	Not sure

ASK ALL
NEW PAGE
Q_OECDACC7b (SINGLE)

**Questions**	**Source**
**Where was this health care professional working?**<br>1 — A primary care centre<br>2 — An outpatient department of a hospital<br>3 — Other<br>4 — Not sure<br>5 — Not applicable	Modified from OECD proposed Set of Questions on Patient Experiences with Ambulatory Care

ASK ALL
NEW PAGE
Q_OECDACC7c (MULTI)

**Questions**	**Source**
**Which of the following best describes the type of care you received?** Please select all the options that apply.<br>1 — Face-to-face consultation in the health care professional's office<br>2 — Telephone consultation<br>3 — Video consultation<br>4 — Home visit<br>5 — Other<br>6 — Not sure	Modified from OECD proposed Set of Questions on Patient Experiences with Ambulatory Care

ASK ALL
NEW PAGE
GPPSACCESS (SINGLE)

**Questions**	**Source**
**How long after initially trying to book the appointment did the appointment take place?**<br>1 — On the same day<br>2 — On the next day<br>3 — A few days and up to a week later<br>4 — More than a week and up to one month later<br>5 — More than one month later<br>6 — Can't remember	Modified from GP Patient Survey (GPPS)

ASK ALL
NEW PAGE
Q_OECDACC9 (SINGLE)

Questions	Source
**Was the time you waited a problem for you?**<br>1 Yes<br>2 No<br>3 Not sure	Modified from OECD proposed Set of Questions on Patient Experiences with Ambulatory Care

ASK ALL
NEW PAGE
Q_OECDACC8 (SINGLE)

Questions	Source
**Did this health care professional spend enough time with you?**<br>1 Yes, definitely<br>2 Yes, to some extent<br>3 No, not really<br>4 No, definitely not<br>5 Not sure	OECD proposed Set of Questions on Patient Experiences with Ambulatory Care

ASK ALL
NEW PAGE
Q_OECDEXP2 (SINGLE)

Questions	Source
**Did this health care professional explain things in a way that was easy to understand?**<br>1 Yes, definitely<br>2 Yes, to some extent<br>3 No, not really<br>4 No, definitely not<br>5 Not sure	OECD proposed Set of Questions on Patient Experiences with Ambulatory Care

ASK ALL
NEW PAGE
Q_GPPSTRUST (SINGLE)

Questions	Source
**Did you have confidence and trust in the health care professional you saw or spoke to?**<br><br>1 — Yes, definitely<br>2 — Yes, to some extent<br>3 — No, not really<br>4 — No, definitely not<br>5 — Not sure	Modified from GP Patient Survey (GPPS)

ASK ALL
NEW PAGE
Q_OECDEXP5 (SINGLE)

Questions	Source
**Overall, how would you rate the quality of this consultation?**<br><br>1 — Excellent<br>2 — Very good<br>3 — Good<br>4 — Fair<br>5 — Poor<br>6 — Not sure	OECD proposed Set of Questions on Patient Experiences with Ambulatory Care

SECTION 9 ‘Your last consultation’ TIMING END

SECTION 10 ‘COVID-19’ TIMING START

ASK ALL
NEW PAGE
The next questions are about if you have tested positive for COVID-19 and symptoms you may have experienced.

Q_COVID1 (SINGLE)

Questions		Source
**Have you ever tested positive for COVID-19 (using a rapid point-of-care test, self-test, or laboratory test) or been told by a doctor or other health care provider that you have or had COVID-19?**		Modified from Household Pulse Survey US
1	Yes	
2	No	
3	Not sure	

ASK IF Q_COVID1 = 1
Q_COVID2 (SINGLE)

Questions		Source
**Did you have any symptoms lasting 2 months or longer that you did not have prior to having coronavirus or COVID-19?** *Long term symptoms may include: tiredness or fatigue, difficulty thinking, concentrating, forgetfulness, or memory problems (sometimes referred to as "brain fog", difficulty breathing or shortness of breath, joint or muscle pain, fast-beating or pounding heart (also known as heart palpitations), chest pain, dizziness on standing, menstrual changes, changes to taste/smell, or inability to exercise.*		Modified from Household Pulse Survey US
1	Yes, symptoms lasted between 2-3 months	
2	Yes, symptoms lasted between 3-6 months	
3	Yes, symptoms lasted between 6 months - 1 year	
4	Yes, symptoms lasted at least 1 year	
5	No	
6	Not sure	

ASK IF Q_COVID2 = 1 TO 4
Q_COVID3 (SINGLE)

Questions		Source
**Do you still have these long-term symptoms?**		Modified from Household Pulse Survey US
1	Yes	
2	No	
3	Not sure	

ASK IF Q_COVID3 = 1

Q_COVID4 (SINGLE)

**Questions**		**Source**
**Do these long-term symptoms reduce your ability to carry out day-to-day activities compared with the time before you had COVID-19?**		Modified from Household Pulse Survey US
1	Yes, a lot	
2	Yes, a little	
3	Not at all	
3	Not sure	

SECTION 10 'COVID-19' TIMING END

SECTION 11 'About yourself' TIMING START

ASK ALL
NEW PAGE

About yourself

Finally, we would like to ask you some information about yourself. It is important to collect this information to understand whether there are any differences in the quality of services experienced by different groups of people.

ASK ALL
NEW PAGE
Q_WB151

**Questions**	**Source**
**How much do you weigh?**<br>1 — ___kilos (MIN 20, MAX 500) (ALTERNATIVE IN IMPERIAL)<br>99 — Don't know<br>97 — Prefer not to say	Modified from WELL-BEING QUESTIONNAIRE FOR PISA 2018 (INTERNATIONAL OPTION)

1	___kilos (MIN 20, MAX 500) (ALTERNATIVE IN IMPERIAL)
99	Don't know
97	Prefer not to say

ASK ALL
NEW PAGE
Q_WB152 (SINGLE)

**Questions**	**Source**
**How tall are you?**	Modified from WELL-BEING QUESTIONNAIRE FOR PISA 2018(INTERNATIONAL OPTION)

1	___centimeters (MIN 50, MAX 250) (ALTERNATIVE IN IMPERIAL)
99	Don't know
97	Prefer not to say

ASK ALL
NEW PAGE
Q_ISCED (SINGLE)

**Questions**	**Source**
**What is the highest educational level that you have attained?**	Modified from International Standard Classification of Education (ISCED)

1	Early childhood education
2	Primary education
3	Lower secondary education
4	Upper secondary education
5	Post-secondary non-tertiary education
6	Short-cycle tertiary education
7	Bachelor's degree or equivalent level
8	Master's degree or equivalent level
9	Doctoral or equivalent level

ASK ALL
NEW PAGE
Q_SEX (SINGLE, OPEN)

Questions	Source
**Which of the following best describes you?**<br><br>1 – Female<br>2 – Male<br>3 – LEAVE BLANK<br>4 – Other<br>97 – Prefer not to say	Modified from OECD Risks That Matter

ASK ALL
NEW PAGE
Q_GENDERID (COUNTRY SPECIFIC QUESTION)

ASK ALL
NEW PAGE
Q_LGBT (SINGLE)

Questions	Source
**This question is about your sexual orientation. Do you identify as:**<br><br>1 – Heterosexual or straight (that is, attracted to the opposite sex)<br>2 – Homosexual (gay or lesbian, that is, attracted to the same sex)<br>3 – Bisexual (attracted to both sexes)<br>4 – Other<br>97 – Prefer not to say	Modified from UK National LGBT Survey

ASK ALL
NEW PAGE
Q_OECDLIT5a (SINGLE)

Questions	Source
**Which of these terms best describes your current work situation?**<br><br>1 – Self-employed [work for yourself]<br>2 – In paid employment [work for someone else]<br>3 – Looking for work<br>4 – Looking after the home<br>5 – Unable to work due to sickness or ill-health<br>6 – Retired<br>7 – Student<br>8 – Not working and not looking for work<br>9 – Apprentice<br>10 – Other<br>99 – Don’t know	OECD Measuring Financial Literacy

ASK ALL
NEW PAGE
Q_OECDLIT7 (SINGLE)

Questions	Source
**Which of these categories does your household net income usually fall into?**<br><br>1 – Up to $X a month<br>2 – Between $X and $Y a month<br>3 – $Y or more a month<br>99 – Don't know<br>97 – Prefer not to say	Modified from OECD Measuring Financial Literacy

ASK ALL
NEW PAGE
Q_MONMED (SINGLE)

Questions	Source
**<u>In the past 12 months</u>, did you have problems paying or were unable to pay any medical bills?**<br><br>1 – Yes<br>2 – No<br>98 – Not applicable	National Health Interview Survey (NHIS)

ASK ALL
NEW PAGE
Q_MON (Show on different screens)

Questions	Source
**How often <u>in the past 12 months</u> would you say you were worried or stressed about the following things?**<br><br>MONMEAL – Having enough money to buy healthy meals?<br>MONRENT – Having enough money to pay your rent or mortgage?<br>MONBILLS – Having enough money to pay for other monthly bills, like electricity, heat, and your telephone?<br><br>Responses (SINGLE)<br>1 – Always<br>2 – Often<br>3 – Sometimes<br>4 – Rarely<br>5 – Never	Modified from 2017 Commonwealth Fund International Health Policy Survey of Older Adults

ASK ALL
NEW PAGE
MEDHIMS6

Questions	Source
**Were you born in (SURVEY COUNTRY)?** (SINGLE, OPEN)<br><br>1 – Yes<br>2 – No (Please state the country you were born in ______________)	Mediterranean Household International Migration Survey (MED-HIMS)

ASK ALL
NEW PAGE
MEDHIMS7

Questions	Source
**Are you a citizen of (SURVEY COUNTRY)?** (SINGLE, OPEN)<br><br>1 – Yes<br>2 – No (Please state what country you are a citizen of ______________)	Mediterranean Household International Migration Survey (MED-HIMS)

ASK ALL
NEW PAGE
Q_OECDLITii

Questions	Source
**Which of these best describes the type of area in which you live?**<br>1 – City<br>2 – Town or suburb<br>3 – Rural area<br>4 – Don’t know	Modified from OECD Measuring Financial Literacy

ASK ALL
NEW PAGE
OECDLIT2a (OPEN)

Questions	Source
**How many children <u>under the age of 18</u> live with you, in your household?**<br>WRITE IN NUMBER (MIN 0, MAX 20)	OECD Measuring Financial Literacy

ASK ALL
NEW PAGE
OECDLIT2b (OPEN)

Questions	Source
**How many people <u>aged 18 and over</u> live with you, in your household? Please do not count yourself**<br>WRITE IN NUMBER (MIN 0, MAX 20)	OECD Measuring Financial Literacy

ASK ALL
NEW PAGE
WHODIS (Show on different screens)

Questions	Source
**Should you need help, how easy is it for you to get help from the following people?**<br><br>WHODIS1 — A close family member (including your partner)?<br>WHODIS2 — Friends, neighbours and co-workers?<br><br>Responses (SINGLE)<br>1 — Very easy<br>2 — Easy<br>3 — Neither easy nor difficult<br>4 — Difficult<br>5 — Very difficult<br>6 — Not applicable	Modified from WHO/ World Bank model-disability-survey

ASK ALL
NEW PAGE
WHOWB11 (SINGLE)

Questions	Source
**Do you need physical care or support, such as help with eating, dressing, bathing, moving around the house or assistance outside the house such as for using transportation?**<br><br>1 — Yes<br>2 — No	Modified from WHO/ World Bank model-disability-survey

ASK ALL
NEW PAGE
Q_WHOWB12 (SINGLE)

Questions	Source
**Do you need emotional care or support, such as comfort, advice or counseling?**<br><br>1 — Yes<br>2 — No	Modified from WHO/ World Bank model-disability-survey

ASK ALL
NEW PAGE
Q_WHOWB13 (SINGLE)

Questions	Source
**Do you need support for health care, such as administering medicines, changing bandages or arranging for appointments with primary care providers?**<br><br>1 — Yes<br>2 — No	Modified from WHO/ World Bank model-disability-survey

ASK ALL
NEW PAGE
Q_TRUST

Questions	Source
**How strongly do you agree or disagree that the health care system can be trusted?**	OECD PaRIS

1	Strongly disagree
2	Disagree
3	Neither agree nor disagree
4	Agree
5	Strongly agree

ASK ALL
NEW PAGE
Q_RESPONDENT

Questions	Source
**Who was the main person or people that filled in this questionnaire?**	NHS Inpatient Survey 2022

1	The person invited to complete the survey
2	A friend or relative of the person invited to complete the survey
3	Both the person invited to complete the survey and a friend or relative together
4	The person invited to complete the survey with the help of a health care professional or care worker

Thank you for taking the time to complete this questionnaire

SECTION 11 ‘About yourself’ TIMING END

Annex A. Source questionnaires of the PaRIS-PQ

Source	Reference
Commonwealth Fund (CWF) Surveys: 2016 IHP, 2017 IHP	The Commonwealth Fund (2016), *International Health Policy Survey*.<br>The Commonwealth Fund (2017), *International Health Policy Survey*.
eHealth Literacy Scale (eHEALS)	Norman CD, Skinner HA (2006a), eHEALS: The eHealth literacy scale. *Journal of Medical Internet Research*, *8*(4).
European Health Interview 2020 Edition	Eurostat and European Commission (2020), *European Health Interview Survey (EHIS wave 3) Methodological manual*. Luxembourg: Publications Office of the European Union.
GP Patient Survey (GPPS)	Campbell J, Smith P, Nissen S, Bower P, Elliott M, Roland M. The GP Patient Survey for use in primary care in the National Health Service in the UK—development and psychometric characteristics. *BMC Fam Pract*. 2009;10:57.<br><br>https://www.gp-patient.co.uk/
Household Pulse Survey US	United States Census Bureau (2023), *Phase 3.7 Household Pulse Survey*. https://www.census.gov/programs-surveys/household-pulse-survey/technical-documentation/questionnaires.2023.html#phase3.7
International Standard Classification of Education (ISCED)	UNESCO Institute for Statistics (2011), *International Standard Classification of Education ISCED 2011*
International Physical Activity Questionnaire - Short Form (IPAQ-SF)	Craig CL, Marshall AL, Sjostrom M, Bauman A, Booth ML, Ainsworth BE, Pratt M, Ekelund U, Yngve A, Sallis JF, Oja P: International Physical Activity Questionnaire: 12-country reliability and validity. *Medicine and Science in Sports and Exercise*. 2003, 35: 1381-1395.
Medicare Patient Engagement Questions	Parker JL, Regan JF, Petroski J (2014), Beneficiary activation in the Medicare population. *Medicare & Medicaid Research Review*, 4, E1-E14.<br><br>https://www.cms.gov/data-research/research/medicare-current-beneficiary-survey
Mediterranean Household International Migration Survey (MED-HIMS)	The European Union (2019), *Mediterranean Household International Migration Survey (MED-HIMS)*.
National Health Interview Survey (NHIS)	Cohen RA, Cha AE (2023), *Problems paying medical bills: United States, 2021.*
NHS Inpatient Survey 2022	National Health Service, Care Quality Commission (2022), *NHS inpatient survey*
OECD Measuring Financial Literacy	OECD INFE (2011) Measuring Financial Literacy: Core Questionnaire in *Measuring Financial Literacy: Questionnaire and Guidance Notes for conducting an Internationally Comparable Survey of Financial literacy.*

	Paris: OECD.
OECD proposed Set of Questions on Patient Experiences with Ambulatory Care	OECD (2018), *OECD Health Working Papers No. 102*.
OECD Risks That Matter	OECD (2018), *OECD Risks That Matter Core Questionnaire*.
Patient-Centered Medical Home (PCMH)	Measures From the CAHPS Patient-Centered Medical Home Item Set. Content last reviewed May 2017. Agency for Healthcare Research and Quality, Rockville, MD. https://www.ahrq.gov/cahps/surveys-guidance/item-sets/pcmh/measures.html
Person-Centred Coordinated Care Experience Questionnaire (P3CEQ)*	Lloyd H, Fosh B, Whalley B, Byng R, Close J (2019), Validation of the person-centred coordinated care experience questionnaire (P3CEQ). *International Journal for Quality in Health Care*, *31*(7), 506-512.<br><br>*The P3CEQ is copyright [2018] University of Plymouth. All rights reserved.
PISA-D Main Study Teacher Questionnaire IS	OECD (2016) *OECD PISA-D Main Study Teacher Questionnaire IS*
Porter-Novelli Consumer Preferences Scale	Maibach EW, Weber D, Massett H, Hancock GR, Price S (2006), Understanding consumers' health information preferences development and validation of a brief screening instrument. *Journal of health communication*, *11*(8), 717-736.
Patient Reported Experiences and Outcomes of Safety in Primary Care (PREOS-PC-6)	Mounce L, Gangannagaripalli J, Ricci-Cabello I, Avery AJ, Valderas JM (2017, October). Development of two short versions of the Patient Reported Experiences and Outcomes of Safety in Primary Care (PREOS-PC) questionnaire: PREOS-PC compact and PREOS-PC-6. *Quality of Life Research* (Vol. 26, No. 1, pp. 110-110).
PROMIS® Scale v1.2 – Global Health	Available from https://www.promishealth.org/57461-2/
PROMIS Adult Short Form v1.0 - Pain Interference	Available from https://www.promishealth.org/57461-2/
PROMIS Item Bank v. 1.0 – Dyspnea	Available from https://www.promishealth.org/57461-2/
UK National LGBT Survey	Department of Education (2018), *National LGBT Research Report*.<br><br>Available from https://www.gov.uk/government/publications/national-lgbt-survey-summary-report
Well-being questionnaire for PISA 2018	OECD (2018), *Well-being questionnaire for Pisa*.
WHO/ World Bank model-disability-survey	World Health Organization (2017) *Model Disability Survey*.<br><br>Available from https://www.who.int/publications/i/item/9789241512862.
WHO Well-being Index (WHO-5)	Heun R, Bonsignore M, Barkow K, Jessen F (2001), Validity of the five-item WHO Well-Being Index (WHO-5) in an elderly population. *European archives of psychiatry and clinical neuroscience*, *251*, 27-31.
