# Supplementary material for: International survey of people living with chronic conditions (PaRIS survey): effects of general practitioner non-participation on the representativeness of the Norwegian patient data
Source: BMC Health Serv Res. 2024 Oct 18;24:1257. doi: 10.1186/s12913-024-11751-0 (PMC11487967; doi:10.1186/s12913-024-11751-0)
Supplement: Supplementary file 2 — Supplementary Material 2. [file 12913_2024_11751_MOESM2_ESM.pdf]

PaRIS

Patient Reported Indicator Surveys

PaRIS Primary Care Practice Questionnaire (PaRIS-PCPQ)

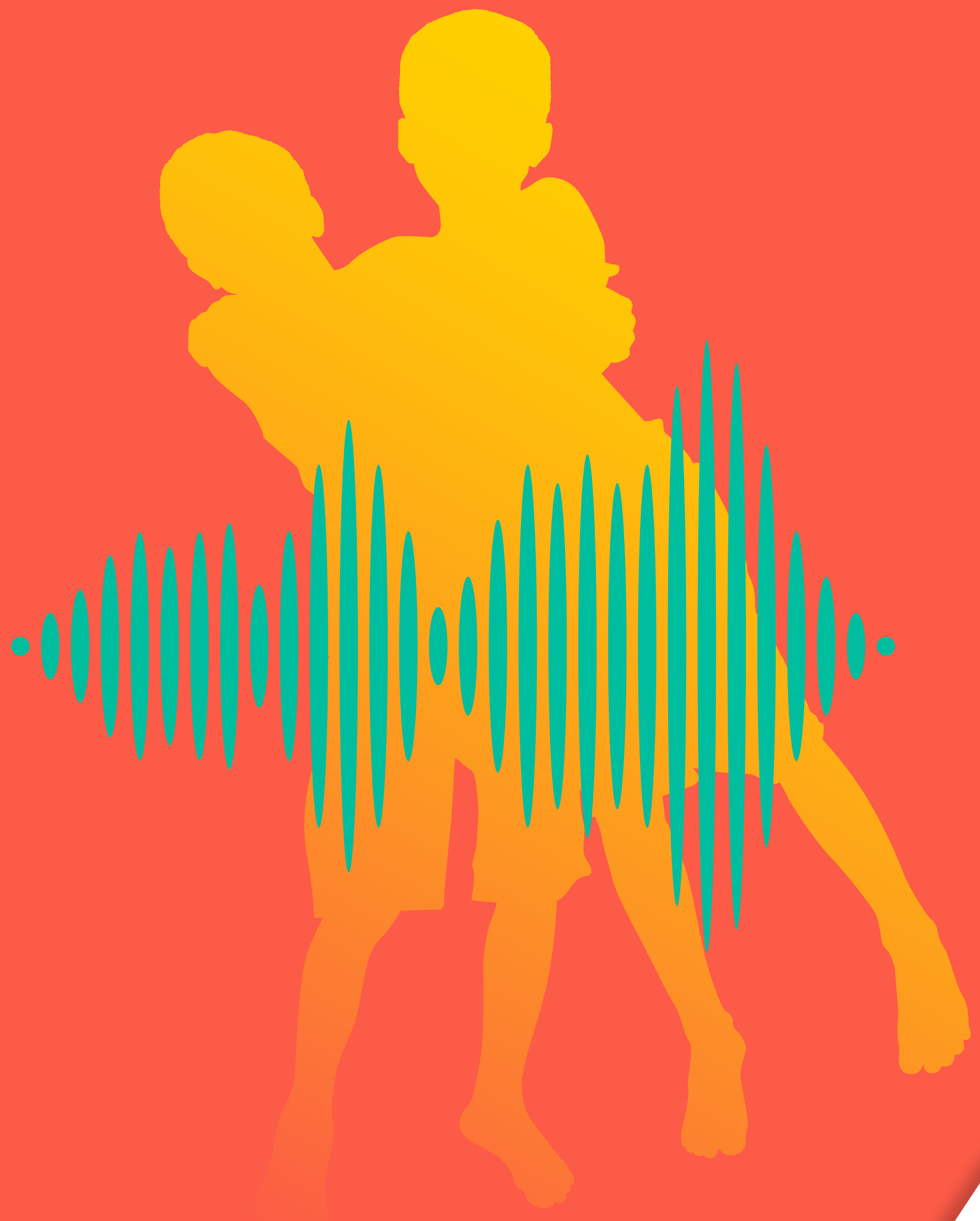

OECD

OECD PaRIS Primary Care Practice Questionnaire (PaRIS-PCPQ)

Version for scripting online survey
2024

© OECD 2024 The use of this work, whether digital or print, is governed by the Terms and Conditions to be found at https://www.oecd.org/termsandconditions.

Table of contents

Background 3
OECD PaRIS-PCPQ for scripting 4
Glossary for terms in the PaRIS-PCPQ 20

Annex A. Source questionnaires of the PaRIS-PCPQ 22

Background

The OECD's Patient-Reported Indicator Surveys (PaRIS) initiative aims to evaluate healthcare outcomes and patient experiences internationally, focusing on primary care performance. The primary tool utilised in this initiative is a questionnaire designed to assess the experiences and outcomes among individuals aged 45 years and older who are treated in primary care. To provide additional context and insights into patient-reported experiences and outcomes, a complementary questionnaire called the Primary Care Practice Questionnaire (PaRIS-PCPQ) is employed. This questionnaire specifically assesses practice characteristics and characteristics of care delivered, with particular emphasis on care for chronic conditions.

The PaRIS-PCPQ was developed based on the PaRIS conceptual framework following a rigorous process of literature review, a modified Delphi process and stakeholder involvement. The questionnaire was translated into the languages of participating countries, subjected to cross-national cognitive testing and pilot-tested in a field trial.

This document comprises the English source version of the PaRIS-PCPQ for scripting an online survey, which was utilised for data collection between 2022 and 2023. Additionally, it provides the sources for the items incorporated into the PaRIS-PCPQ.

The PaRIS Initiative (Patient-Reported Indicator Surveys) of the OECD aims to develop and implement internationally comparable patient-reported indicators, more specifically patient-reported outcome measures (PROMs) and patient-reported experience measures (PREMs).

The OECD developed the PaRIS survey instruments with an international consortium of Nivel, Ipsos, University of Exeter, Optimedis AG and Avedis Donabedian Research Institute.

OECD PaRIS-PCPQ for scripting

Note to the reader: The questionnaire for PC providers was administered online only. Accordingly, the questionnaire has a layout as a version for scripting, rather than a paper-based version. Please note following elements for clarification:

- ASK ALL: item is applicable to all respondents
- ASK IF: applicability of the item is conditional on another item
- (SINGLE): items where respondents can give one of the response options
- (MULTI): items where respondents may tick multiple response options
- (LOOP): items consisting of multiple questions, each with the same answering categories
- Terms marked with a * are defined in a glossary at the end of this document

Thank you for your help with this important research.

This survey is being conducted by <<RESEARCH ORGANISATION>> on behalf of <<NAME OF LEAD ORGANISATION IN COUNTRY>>. It is part of an international survey led by the Organisation for Economic Co-operation and Development (OECD).
The survey aims to understand how your practice manages patients with chronic conditions. It is also being conducted in other countries across the OECD area and will be used to compare how different health care systems work. Participating in this survey will help to strengthen the health care system to better respond to the needs of people with chronic conditions.
The survey is designed to be completed by primary care physicians, nurses, or other allied health care professionals (such as physiotherapists) or practice managers. The questionnaire should be completed by persons who are most familiar with how the practice is organised and operates.
All questions refer to the practice you work in and the current point in time. The term "practice" refers to the practice (solo practice or group practice) or health center or other facility you work in. Answers should reflect as much as possible the views and practices of the entire primary health care medical team.
It should take you about 10 to 15 minutes to take part.
Taking part is voluntary and your answers will be completely confidential. Your answers will be combined with those from other practices and there will be nothing in the results that could identify you.
If you have any queries about the questionnaire, please call our helpline number on [LOCAL NUMBER] or email [LOCAL EMAIL].

CONSENT (SINGLE CODE)
Do you agree to take part in the survey?

Yes CONTINUE
No CLOSE

SECTION 1 'About your practice' TIMING START

ASK ALL
NEW PAGE

**Questions**	**Source**
**First, we would like to ask you some questions about the practice you work in.**<br>LOCATION (SINGLE)<br>**Where is the practice you work located?**	Adapted from Commonwealth Fund

1	City
2	Town or suburb
3	Rural area
4	Not sure

ASK ALL
NEW PAGE

**Questions**	**Source**
PRACTICETYPE (SINGLE)<br>**What type of practice do you work in?**	Adapted from PCAT

1	Solo practice (a practice that is run by a single physician or health care professional)
2	Group practice with own patients (two or more physicians or health care professionals who share premises but do not share a common pool of patients)
3	Group practice with shared patients (two or more physicians or health care professionals who share a common pool of patients)
4	Multi-specialty group practice (a practice that is run by two or more physicians or health care professionals who have different specialisations)
5	Other
6	Not sure

ASK ALL
NEW PAGE
SERVICES (SINGLE)

**Questions**	**Source**
**Does the practice you work in offer medical services to patients without an appointment?**	Adapted from Québec Survey

1	Yes
2	No
3	Not sure

ASK IF SERVICES =1
NEW PAGE
SERVICES_OPTIONS (MULTI)

Questions	Source
**Which types of patients do you offer medical services to without an appointment?**<br>Select all the options that apply.<br>1 – Patients with **urgent** medical problems **who are registered** at the practice<br>2 – Patients with **non-urgent** medical problems **who are registered** at the practice<br>3 – Patients with **urgent** medical problems **who are not registered** at the practice<br>4 – Patients with **non-urgent** medical problems **who are not registered** at the practice	Adapted from Québec Survey

ASK ALL
NEW PAGE
CLOSED_OPTIONS (MULTI)

Questions	Source
**Which of the following out-of-hours options does your practice provide to your patients? Please do not include services commissioned outside the practice, such as XXX. [SUBJECT TO LOCALISATION TO SPECIFY SERVICES commissioned outside the practice, if applicable]**<br>Please select all the options that apply.<br>1 – Visiting before or after practice's regular working hours [LOCALISATION TO SPECIFY "REGULAR WORKING HOURS" in the country] (at least once per week)<br>2 – Visiting the practice on a weekend day<br>3 – Directly contacting a physician or nurse via remote options (e.g., telephone, online chat/messaging)<br>4 – Leaving a message on an answering machine, writing an e-mail, or completing an online form with a return call from a physician or nurse shortly afterwards<br>5 – Other<br>6 – None of these<br>7 – Not sure	Adapted from Commonwealth Fund, QUALICOPC and PCAT

ASK ALL
NEW PAGE
HOMEVISITS (SINGLE)

Questions	Source
**Does at least one physician or other health care professional from the practice make home visits?**	Adapted from Québec survey

1	Yes
2	No
3	Not sure

ASK ALL
NEW PAGE
ROLE_STAFF (MULTI)

Questions	Source
**What are the roles and functions of the staff (other than physicians) working in your practice in chronic care management?**<br>Please select all the options that apply.	Adapted from Québec survey

1	Patient education and counselling (e.g., on tobacco use, diet, or physical activity)
2	Chronic disease management tasks (e.g., diabetes control, wound care, or measuring blood pressure)
3	Other roles and functions, please specify ________________
4	There are no additional staff (other than physicians) in the medical team
5	Not sure

ASK ALL
NEW PAGE
ONLINE (MULTI)

Questions	Source
**Does your practice offer phone, video, or other online methods for consultations to patients?**<br>Please select all the options that apply.	OECD PaRIS

1	Phone consultations
2	Video consultations
3	Consultations using other remote options (e.g., e-mail, text message, messaging through patient platforms)
4	None of these
5	Not sure

ASK ALL
NEW PAGE
TIME (LOOP)

Questions	Source
**How much time is scheduled in your practice for each of the following actions (performed by physicians)?**	Adapted from Québec survey

A	Consultations for a newly registered patient
B	Regular and follow-up consultations
C	Urgent consultations
D	Home visits (excluding travel time)
ASK E IF ONLINE = 1	
E	Phone consultations
ASK F IF ONLINE = 2	
F	Video consultations
ASK G IF ONLINE = 3	
G	Consultations using other remote options (e.g., e-mail, text message, messaging through patient platforms)

Responses (SINGLE)

1	Up to 5 minutes
2	6-10 minutes
3	11-15 minutes
4	16-20 minutes
5	21-30 minutes
6	More than 30 minutes
7	Not pre-specified
8	Not sure
9	Does not apply

ASK ALL
NEW PAGE
FUNDING_MODEL (MULTI)

Questions	Source
**How are physicians paid in your practice?**<br>Please select all the options that apply.	Adapted from Québec survey

1	Fee for service*
2	Sessional fees *
3	Fixed salary*
4	Pay for performance*
5	Capitation fee *
6	Other
7	Not sure

*Defined in the glossary

ASK ALL
NEW PAGE
ADD_PAYMENTS (MULTI)

Questions	Source
**Does your practice receive dedicated payments in the following circumstances?**<br>Please select all the options that apply.<br><br>1 – Achievement of targets for screening or prevention<br>2 – Meeting targets for referring patients<br>3 – Having disadvantaged patients (e.g., individuals who are socially unprotected and/or financially vulnerable) in your practice<br>4 – Working in a specific area (rural or disadvantaged)<br>5 – Achievement of other targets related to cost containment<br>6 – Achievement of clinical or health process or outcome indicators<br>7 – Management of patients with specific chronic conditions (e.g., diabetes, COPD) by bundled payment<br>8 – Management of patients with specific chronic conditions (e.g., diabetes, COPD), not by bundled payment<br>9 – [should remain blank]<br>10 – Other (PLEASE WRITE IN________________)<br>11 – None of these<br>12 – Not sure	Adapted from QUALICOPC

SECTION 1 ‘About your practice’ TIMING END
SECTION 2 ‘Organisation of care’ TIMING START

ASK ALL
NEW PAGE

The next questions are about the organisation of care in your practice.

FORMAT_RECORDS (MULTI)

Questions	Source
**In which formats do you keep patient medical records* in your practice?**<br>Please select all the options that apply.<br><br>1 – Medical records are kept electronically<br>2 – Medical records are kept in paper format<br>3 – Not sure<br>*Defined in the glossary	Adapted from QUALICOPC

ASK ALL
NEW PAGE
KEPT_RECORDS (MULTI)

Questions	Source
**For which types of patients are medical records kept in your practice?** Please select all the options that apply.	Adapted from QUALICOPC

1	Medical records are kept for non-regular attending patients
2	Medical records are kept for regular attending patients
3	Medical records are kept for registered patients
4	Medical records are kept for all patients
5	Not sure

ASK ALL
NEW PAGE
INFO_RECORDS (MULTI)

Questions	Source
**Which, if any, of the following information is normally included in patients' medical record?** Please select all the options that apply.	Adapted from QUALICOPC

1	Reasons for the consultation
2	Family medical history
3	Living situation (alone, partner, family)
4	Employment situation
5	Ethnicity
6	Smoking habit
7	Substance use (alcohol and/or drug use)
8	Weight and height
9	Clinical parameters (such as blood pressure)
10	Test results
11	Diagnosis
12	Prescribed medications
13	Other information
14 TO 17	[should remain blank]
18	Not sure

ASK ALL
NEW PAGE
AVAIL_RECORDS (SINGLE)

Questions	Source
**Are medical records available when patients are seen?**	Adapted from PCAT

1	Yes, always
2	Yes, sometimes
3	No
4	Not sure

ASK ALL

NEW PAGE

PREV_RECORDS (MULTI)

Questions	Source
**When new patients join your practice, do you receive their medical records from their previous practice?**<br>Please select all the options that apply.	Adapted from QUALICOPC

1	Yes, they can be accessed electronically without directly requesting them
2	Yes, when we request them from the previous practice
3	Yes, when the patient brings them
4	No
5	Not sure

ASK ALL

NEW PAGE

COMPUTER_PROCESS (MULTI)

Questions	Source
**Can your practice produce the following information about the patients in your practice using a computerized system (e.g., clinical information system*)?**<br>Please select all the options that apply.	Adapted from Commonwealth Fund

1	List of patients by diagnosis
2	List of patients who are due or overdue for tests or preventive care (e.g., flu vaccine due)
3	List of patients taking a particular medication which have been prescribed in **your practice**
4	List of patients taking a particular medication prescribed **outside** your practice
5	List of all laboratory results for an individual patient ordered by **your practice**
6	List of all laboratory results for an individual patient ordered **outside** your practice
7	A clinical summary of each visit
8	None of this information can be produced using a computerized system
9	Not sure

*Defined in the glossary

ASK ALL
NEW PAGE
COMPUTER_ROUTINE (MULTI)

**Questions**	**Source**
**Which, if any, of the following tasks are routinely performed in your practice using a computerized system (e.g., a clinical information system*)?**<br>Please select all the options that apply.	Adapted from Commonwealth Fund

1	Booking appointments
2	Patients are sent reminder notices when it is time for regular preventive or follow-up care (e.g., flu vaccine or Glycated Haemoglobin for diabetic patients)
3	Orders for laboratory tests are tracked until results reach the practice
4	Health care professionals receive an alert or prompt to provide patients with test results when they reach the practice
5	Health care professionals receive a reminder for guideline-based interventions and/or screening tests
6	None of these
7	Not sure

*Defined in the glossary

ASK ALL
NEW PAGE
INFO_EXCHANGE (MULTI)

**Questions**	**Source**
**Can your practice electronically exchange any of the following information with any health care professionals outside the practice?**<br>Please select all the options that apply.	Adapted from Commonwealth Fund

1	Patient clinical summaries
2	Laboratory and diagnostic test results
3	Electronic medical records
4	None of this information can be exchanged with health care professionals outside the practice
5	Not sure

ASK ALL
NEW PAGE
REVIEW_DATA (MULTI)

**Questions**	**Source**
**Does your practice review indicators on the following aspects of your patients' care?**<br>Please select all the options that apply.	Adapted from Commonwealth Fund

1	Clinical outcomes (e.g., percent of diabetics or asthmatics with good control)

2	Patient satisfaction or patient experiences with care, collected through surveys	
3	Patients' hospital admissions or emergency department use	
4	Percent of patients who have received recommended preventive care (e.g., immunizations, faecal occult blood test, cervical screening test, etc.)	
5	Adherence to prescribed drugs	
6	None of this information is reviewed	
7	Not sure	

SECTION 2 'Organisation of care' TIMING END
SECTION 3 'Chronic care management' TIMING START
ASK ALL
NEW PAGE
The following questions are about chronic care management and the follow-up of patients in your practice.

ASK ALL
NEW PAGE
FOLLOW_UP (MULTI)

Questions		Source
**Do you provide care in your practice (e.g., regular appointments, tests, and reviews) for any of the following chronic conditions?** Please select all options that apply.		Adapted from Québec survey
A	Cardiovascular conditions	
B	Diabetes (type 1 or 2)	
C	Arthritis or other musculoskeletal conditions	
D	Respiratory conditions (e.g., Asthma or COPD)	
E	Alzheimer's disease or other cause of dementia	
F	Mental health conditions (e.g., Depression, anxiety, bipolar disorder, schizophrenia)	
G	Neurological conditions (e.g., epilepsy or migraine)	
H	Chronic kidney disease	
I	Chronic liver disease	
J	Cancer	
K	Other	

ASK ALL
NEW PAGE
COORDINATE_CARE (LOOP)

Questions		Source
**How prepared is your practice to coordinate care with other health and social care providers for the following groups of patients?**		Adapted from Commonwealth Fund
A	Patients with one or multiple chronic condition(s)	
B	Patients with severe mental health problems, such as depression, bipolar disorder, or schizophrenia	
C	Patients with issues of addiction/substance misuse	
D	Patients in need of palliative care	

E	Patients in need of coordination of long-term care (e.g., home-based support services such as nursing or personal care)	
F	Patients in need of coordination of social services in the community (e.g., housing, meals, and transportation)	
G	Patients in need of translation service during consultations	

Responses (SINGLE)

1	Well prepared
2	Somewhat prepared
3	Not prepared
4	Not sure
5	Does not apply

ASK ALL

NEW PAGE

MANAGEMENT (MULTI)

Questions	Source
**Who is in charge of coordinating care*** **for patients with chronic conditions at your practice?**<br>Please select all the options that apply.	Adapted from PCMH-A

1	Care coordination is provided by a health care professional in the practice team
2	Care coordination is provided by a health care professional outside the practice
3	Care coordination is provided by specialized external services
4	Care coordination services are provided in another way
5	Care coordination services are not available
6	Not sure

*Defined in the glossary

ASK ALL

NEW PAGE

CARE_PLANS_PATIENTS (MULTI)

Questions	Source
**Are patient care plans* routinely developed at your practice for any of the following patient groups?**<br>Please select all the options that apply.	OECD PaRIS

1	Patients with any chronic conditions
2	Patients with specific chronic conditions
3	Patients with specific needs (e.g., patients with nutritional needs or complex care needs)
4	Other patients
5	Patient care plans are not routinely developed
6	Not sure

ASK if CARE_PLANS_PATIENTS (MULTI) =1,2,3,4
CARE_PLANS_USE (MULTI) N

You said that patient care plans are routinely developed at your practice for the following patient groups:

INSERT RESPONSE CODES FROM CARE_PLANS_PATIENTS – CODES 1-4 LISTED AS RELEVANT

Questions		Source
**To what extent are care plans being used (i.e., discussed, agreed, updated) in appointments with these patient groups?**		OECD PaRIS
1	They are used with all patients, at least once a year	
2	They are used with all patients, but less than once a year	
3	They are used with some patients, at least once a year	
4	They are used with some patients, but less than once a year	
5	They are not used in consultation with patients	
6	Not sure	

NEW PAGE
ASK IF CARE_PLANS_PATIENTS = 1,2,3,4
CARE_PLANS_DEVELOPED (MULTI)

Questions		Source
**Which of the following are typically involved in the development of patient care plans*?** Please select all the options that apply.		Adapted from ACIC
1	Patients	
2	Patient's caregivers and/or family members	
3	Health care professionals working at your practice	
4	Health care professionals working outside your practice	
5	Someone else	
6	Not sure	

NEW PAGE
ASK IF CARE_PLANS_PATIENTS = 1,2,3,4
CARE_PLANS_INCLUDED (MULTI)

Questions		Source
**Which of the following are typically included in the patient care plans* developed at your practice?** Please select all the options that apply.		OECD PaRIS
1	Summary of patient's medical history	
2	Patient's diagnoses	
3	Patient's medication	
5	Clinical goals (e.g., monitoring/control/reduce blood pressure)	

6	Patient's personal goals (e.g., going on a trip or and maintaining hobbies)	
7	Self-management tasks (e.g., improve/reinforce physical exercise)	
8	Contact details of caregivers and/or family members	
9	Documentation of other involved care providers	
10	Visits scheduled with other care providers (including outside your practice)	
11	Other	
12	Not sure	

NEW PAGE
ASK IF CARE_PLANS_PATIENTS = 1,2,3,4
CARE_PLANS_SHARED (MULTI)

Questions	Source
**Are patient care plans shared with any of the following at your practice (either electronically or on paper)?** Please select all the options that apply.	OECD PaRIS

1	Patients
2	Patient's caregivers and/or family members
3	Health care professionals working at your practice
4	Health care professionals working outside your practice
5	Someone else
6	Not sure

ASK ALL
NEW PAGE
SELF_MANAGEMENT (MULTI)

Questions	Source
**Which of the following statements describe how self-management support* is provided to patients with chronic conditions?** Please select all the options that apply.	Adapted from PCMH-A

1	Self-management support is provided by verbal information during or after the consultation
2	Self-management support is provided by distributing information (pamphlets, booklets, internet/web-based information)
3	Self-management support is provided by referral to self-management classes or educators
4	Self-management support is provided by explicit goal setting and action planning with members of the practice team
5	Self-management support is provided by members of the practice team trained in patient empowerment and problem-solving methodologies
6	Self-management support is not provided in any of these ways
7	Not sure

*Defined in the glossary

ASK ALL
NEW PAGE
GOALS (SINGLE)

Questions	Source
**How often are the self-management goals of your patients with chronic conditions recorded in their medical records?**<br><br>1 – Always<br>2 – Often<br>3 – Sometimes<br>4 – Rarely<br>5 – Never<br>6 – Not sure	Adapted from Commonwealth Fund

ASK ALL
NEW PAGE
WRITTEN (SINGLE)

Questions	Source
**How often are your patients with chronic conditions given written instructions (either electronically or on paper) about how to manage their own care at home (e.g., instructions on what to do to control symptoms, prevent flare-ups, or monitor their condition at home)?** Note: Do not include prescriptions.<br><br>1 – Always<br>2 – Often<br>3 – Sometimes<br>4 – Rarely<br>5 – Never<br>6 – Not sure	Adapted from Commonwealth Fund

ASK ALL
NEW PAGE
REFERRAL (LOOP)

Questions	Source
**To what extent are referral letters (including details on provisional diagnosis and possible test results) written in your practice when patients are referred to another medical specialist?**<br><br>A – Electronic referral letters<br>B – Referral letters on paper<br><br>Responses (SINGLE)<br>1 – Always<br>2 – Often<br>3 – Sometimes	Adapted from Québec survey

4	Rarely		
5	Never		
6	Not sure		

ASK ALL
NEW PAGE
SPECIALIST (LOOP)

Questions	Source
**After the follow-up visit to another specialist, how often does the practice receive timely referral information from this specialist in the following ways?**	Adapted from PCAT

A	Electronic referral letters
B	Referral letters on paper

Responses (SINGLE)

1	Always
2	Often
3	Sometimes
4	Rarely
5	Never
6	Not sure
7	Does not apply

ASK ALL
NEW PAGE
Finally, we would like to ask you some information about yourself.

ASK ALL
NEW PAGE
PROFESSION (SINGLE)

Questions	Source
**What is your professional background?**	Adapted from PCAT

1	Physician in family medicine
2	Physician in general internal medicine
3	Physician in general internal medicine with sub-specialty focus
4	Physician in specialist medical training
5	Nurse practitioner
6	Nurse practitioner specialized in family medicine
7	Practice nurse
8	Physician's assistant
9	Allied health care professional, such as a physiotherapist, dietician, or podiatrist
10	Practice manager (non-medical)
11	Other

Thank you for taking part.

SECTION 3 ‘Chronic care management TIMING END

Glossary for terms in the PaRIS-PCPQ

Capitation fee is a form of remuneration for medical services, which pays for the services provided during a certain period of time as a blanket sum per patient or insured person or per treatment case.

Care coordination is a patient-centered approach to synchronize the delivery of a patient's health care from multiple providers and specialists while helping them to navigate effectively and efficiently through the health care system.

Clinical information system (CIS) is a computer-based system that is designed for collecting, storing, using and making clinical information available to the health care delivery process.

Fee for service is a form of remuneration in which each individual medical service is evaluated and rewarded, in contrast to the flat rate per case or complex service remuneration.

Fixed salary is a fixed amount of remuneration from which taxes and social security are directly deducted before the person receives it.

Guideline-based interventions are based on clinical guidelines which aim to give recommendations on how to diagnose and treat a medical condition. Clinical guidelines summarize the current medical knowledge, weigh the benefits and harms of diagnostic procedures and treatments, and give specific recommendations based on this information. They should also provide information about the scientific evidence supporting those recommendations.

Health care provider is the primary/ambulatory care facility (e.g., practice, clinic) in which ambulatory health care services are provided to the community by health care professionals working either single-handedly or within a team of **health care professionals** (e.g., medical doctors, nurse practitioners, physician assistants, certified nurse specialists trained in a particular field, pediatric or diabetic nursing, certified nurse midwives, clinical social worker, physical therapists).

Immunizations can be rendered as vaccinations.

Medical record is a record of a patient's medical information (as medical history, care or treatments received, test results, diagnoses, and medications taken).

Out-of-hour arrangements give patients the opportunity to see a doctor or nurse of another practice without going to the hospital emergency room or department

Patient care plan is an agreement between the patient and health care professionals detailing the type of health care the patient receives and how the care will be given over an extended period of time (written document).

Patient empowerment refers to a process through which people gain greater control over decisions and actions affecting their health.

Pay for performance in health care comprises payment model that attach financial incentives/disincentives to provider performance in relation to metric-driven outcomes, best practices or patient satisfaction/experience.

Prevention includes a wide range of activities and targeted measures to prevent diseases or threats to health, to reduce the risk of the disease or to delay its occurrence. Preventive measures can be classified as primary, secondary or tertiary prevention according to the time at which they are applied.

Screening: The examination to detect health risks or problems in usually asymptomatic individuals to detect those with a high probability of having or developing a given disease, typically by means of an inexpensive diagnostic test.

Self-management is the active participation by a patient in his or her own health care decisions and interventions. With the education and guidance of professional caregivers, the patient promotes his or her own optimal health or recovery.

Self-management support includes a portfolio of techniques and tools that help patients take a more active role in the management of their condition, including self-care monitoring

lifestyle change, communicating with healthcare professionals, dealing with the consequences of a chronic condition in daily life, etc.

Sessional fees comprise payment mechanisms where a payment is linked to the completion of a time—limited therapeutic or counselling session (this payment model is frequently used in the field of mental health care or rehabilitation).

Annex A. Source questionnaires of the PaRIS-PCPQ

Source Questionnaire	Reference
**QUALICOPC** (Quality and Costs of Primary Care)	Schäfer et al. QUALICOPC, a multi-country study evaluating quality, costs and equity in primary care. BMC Fam Pract. 2011;12:115.
**ACIC** (Assessment of Chronic Illness Care)	Bonomi AE, Wagner EH, Glasgow RE, VonKorff M. Assessment of chronic illness care (ACIC): a practical tool to measure quality improvement. Health Serv Res. 2002;37(3):791-820.
**PCMH-A** (Patient-centered Medical Home Assessment)	MacColl Center for Health Care Innovation, Group Health Research Institute. Version 4.0 (2014) (http://www.improvingchroniccare.org/index.php?p=PCMH-A&s=319)
**Québec Survey**	Levesque JF, Pineault R, Provost S, et al. Assessing the evolution of primary healthcare organizations and their performance (2005-2010) in two regions of Québec province: Montréal and Montérégie. BMC Fam Pract. 2010;11:95.
**PCAT** (Primary Care Assessment Tool, short version)	Shi, L., Starfield, B., Xu, J. Validating the adult primary care assessment tool. J Fam Pract. 2001; 50(2):161-164.
**Commonwealth Fund International Survey of Primary Care Physicians in 10 Nations**	International Survey of Primary Care Doctors (2015) (https://www.commonwealthfund.org/publications/surveys/2015/dec/2015-commonwealth-fund-international-survey-primary-care-physicians)
